# Genotoxic stress in constitutive trisomies induces autophagy and the innate immune response via the cGAS-STING pathway

**DOI:** 10.1038/s42003-021-02278-9

**Published:** 2021-07-02

**Authors:** Maria Krivega, Clara M. Stiefel, Sahar Karbassi, Line L. Andersen, Narendra K. Chunduri, Neysan Donnelly, Andreas Pichlmair, Zuzana Storchová

**Affiliations:** 1 Molecular Genetics, University of Kaiserslautern, Kaiserslautern, Germany; 2 Institute of Virology, TU Munich, München, Germany; 3 Max Planck Institute of Biochemistry, Martinsried, Germany; 4 German Center for Infection Research (DZIF), Munich partner site, München, Germany

**Keywords:** Chromosomes, Chromosomes, Stress signalling

## Abstract

Gain of even a single chromosome leads to changes in human cell physiology and uniform perturbations of specific cellular processes, including downregulation of DNA replication pathway, upregulation of autophagy and lysosomal degradation, and constitutive activation of the type I interferon response. Little is known about the molecular mechanisms underlying these changes. We show that the constitutive nuclear localization of TFEB, a transcription factor that activates the expression of autophagy and lysosomal genes, is characteristic of human trisomic cells. Constitutive nuclear localization of TFEB in trisomic cells is independent of mTORC1 signaling, but depends on the cGAS-STING activation. Trisomic cells accumulate cytoplasmic dsDNA, which activates the cGAS-STING signaling cascade, thereby triggering nuclear accumulation of the transcription factor IRF3 and, consequently, upregulation of interferon-stimulated genes. cGAS depletion interferes with TFEB-dependent upregulation of autophagy in model trisomic cells. Importantly, activation of both the innate immune response and autophagy occurs also in primary trisomic embryonic fibroblasts, independent of the identity of the additional chromosome. Our research identifies the cGAS-STING pathway as an upstream regulator responsible for activation of autophagy and inflammatory response in human cells with extra chromosomes, such as in Down syndrome or other aneuploidy-associated pathologies.

## Abstract (web summary)

Studying trisomic cell lines derived from RPE1 and HCT116 cells, Krivega et al find that autophagy is induced independently of mTORC1 in these cells. Rather, they observe that nuclear accumulation of TFEB and IRF3 and activation of the inflammatory response and autophagy in trisomic cells is dependent on the cGAS-STING pathway.

## Introduction

Whole-chromosomal aneuploidy (hereafter aneuploidy), which results from chromosome segregation errors, is often detrimental to eukaryotic organisms. In humans, organismal aneuploidy interferes with normal development, and only a low percentage of embryos with trisomies of chromosomes 8, 13, 18, and 21 (Warkany, Patau, Edwards, and Down syndrome, respectively) result in live births, although rarely^1^. Infants born with these syndromes suffer from multiple pathologies and have a shortened life expectancy. In somatic cells, missegregating cells are often arrested in the cell cycle or die; even if they escape this fate, they are outgrown by normal cells due to the proliferative disadvantage associated with aneuploidy, or they may be actively removed by immune cells^2–5^. At the same time, aneuploidy provides an advantage in stressful, variable environmental conditions^6,7^. Aneuploidy is also a common feature in nearly 90% of solid tumors. Here, the degree of aneuploidy correlates positively with invasiveness, resistance to chemotherapy, immune evasion, and poor patient prognosis^8^. The molecular mechanisms underlying the striking and variable consequences of aneuploidy remain poorly understood.

Recently established model mammalian somatic cell lines with defined chromosome gains have allowed in vitro studies of the effects of aneuploidy on cellular function^9–11^. These studies revealed that the gain of a single chromosome triggers a uniform and conserved deregulation of specific pathways that primarily affect cell proliferation and genome and proteome homeostasis^7,12,13^. These consequences on cellular physiology are referred to as aneuploidy-associated stresses and are thought to occur largely due to the overexpression of proteins from the extra chromosomes, which overwhelms the cellular ability to maintain protein homeostasis (proteostasis)^7,13–16^. As a result, cells with extra chromosomes exhibit impaired protein folding, cytoplasmic foci of aggregated proteins, increased proteasomal activity, and sensitivity to conditions that disrupt proteostasis^14,16,17^. In addition, aneuploid cells suffer from genomic instability characterized by replication stress, accumulation of DNA damage, and increased chromosomal aberrations^3,18–20^.

Aneuploid model cell lines have also been shown to activate the type I interferon response, but the upstream triggers have not been identified^21,22^. Interestingly, DNA damage and replication stress have recently emerged as potential triggers of the inflammatory response in mammalian cells^23–26^. Here, cGAS (cyclic guanosine monophosphate (GMP)–adenosine monophosphate (AMP) synthase) recognizes cytosolic DNA, which catalyzes the conversion of GTP and ATP into the second messenger cyclic GMP–AMP (2′3′-cGAMP)^27^. The latter serves as a ligand for the adapter protein stimulator of interferon (IFN) genes (STING), which dimerizes after cGAMP binding and translocates from the ER to the Golgi apparatus. Activated STING recruits and activates TANK-binding kinase 1 (TBK1) and I kappa B kinase (IKK), which in turn phosphorylates transcription factors IRF3 and NF-κB, respectively^28^. Phosphorylated IRF3 and NF-κB translocate to the nucleus, where they trigger transcription of type 1 interferons and other cytokines, which subsequently elicit the expression of interferon-stimulated genes (ISGs)^29^. Whether this signaling pathway is activated in constitutive trisomic cells has not yet been tested.

Cells with extra chromosomes also upregulate autophagy and lysosomal genes and show increased levels of LC3-positive autophagosomes^11,21^. Moreover, murine aneuploid cells were selectively sensitive to treatment with chloroquine, a drug that impairs lysosomal function and thus blocks autophagic degradation^17^. These findings suggest that autophagic activity is constitutively increased in aneuploid cells, likely to attenuate the deleterious effects of aneuploidy-associated stresses. Autophagy is a conserved, tightly regulated cellular mechanism that degrades unnecessary or dysfunctional cytoplasmic components and allows recycling of their constituents^30^. During autophagy, the phagophore membrane carrying the lipidated form of the LC3 protein (LC3-II) engulfs the cytoplasmic components to be degraded, and subsequently fuses with lysosomes to enable degradation. Under nutrient-rich conditions, activated mTORC1 kinase, a key nutrient sensor, suppresses autophagosome assembly by inhibitory phosphorylation of ULK1 and other proteins^31^. Starvation inactivates mTORC1, abrogating the inhibitory phosphorylations^32^. Other mTORC1 targets include the transcription factors of the MIT/TFEB family, which regulate the expression of autophagy- and lysosome-specific genes^33,34^. In fed conditions, active mTORC1 phosphorylates TFEB to sequester it in the cytoplasm, whereas during starvation, when mTORC1 is inactive, TFEB translocates to the nucleus and activates transcription of its targets^35^, thereby facilitating autophagy and lysosomal degradation^36^. Autophagy is also activated by other cellular insults, such as oxidative stress or the unfolded protein response^31^. Recently, autophagy was shown to be activated by the innate immune response pathway in the context of genomic instability^37^. While autophagy is regulated by multiple signaling pathways, their individual contribution to increased autophagy and lysosomal activity in cells with extra chromosomes remain enigmatic.

Building on our previous knowledge of the consequences of chromosome gain in mammalian cells and recent findings that the activation of both autophagy and the innate immune response might be tightly linked, we sought to determine the cellular mechanisms that contribute to the constitutive upregulation of autophagy and lysosomal pathways in trisomic cells. To this end, we used a series of trisomic cell lines derived from RPE1 (a diploid, immortalized retinal pigmented epithelial cell line) and HCT116 (a diploid colorectal cancer cell line) generated by microcell-mediated chromosome transfer^11^. Strikingly, we show that activation of autophagy and the lysosomal pathway in constitutively trisomic cells is independent of mTORC1 status. Instead, the cGAS–STING pathway is constitutively active in trisomic cells and triggers type I interferon response and ISG expression, as well as transcriptional upregulation of autophagy and lysosomal genes. Importantly, we demonstrate that cGAS–STING-dependent activation of the inflammatory response and autophagy also occurs in human primary embryonic fibroblasts isolated from embryos with trisomy syndromes. Taken together, our results reveal the mechanism of autophagy activation in response to chromosome gain and extend the link between autophagy and innate immune signaling.

## Results

### TFEB-dependent transcription and autophagy is activated in constitutive trisomies

**Fig. 1:**
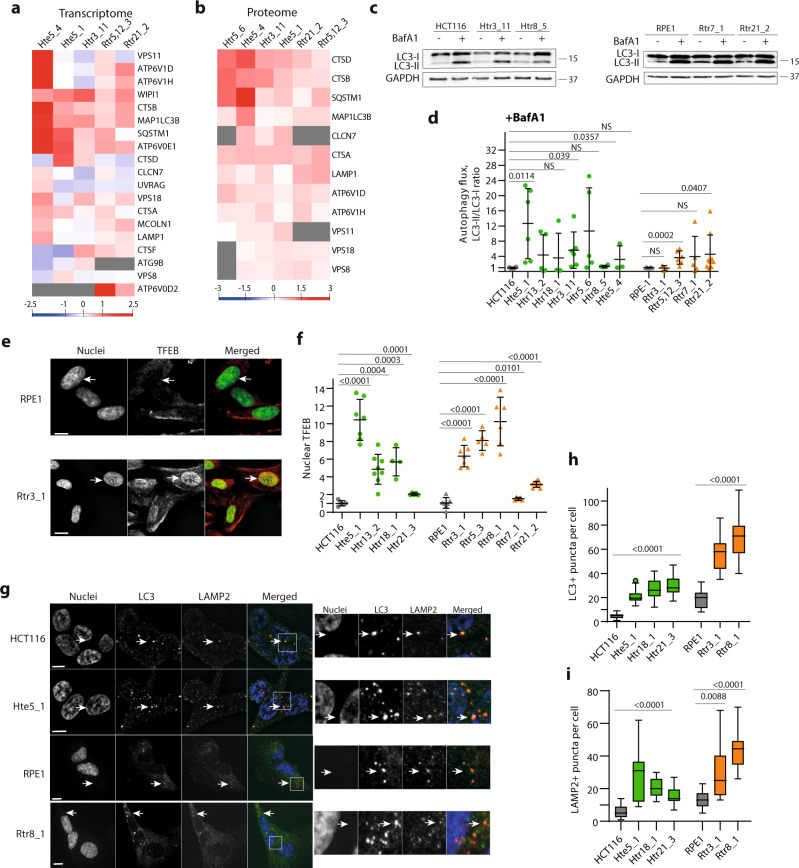
Autophagy and lysosomal degradation is activated in cells with extra chromosomes. **a** Transcriptome and **b** proteome analysis of TFEB targets. The heat maps show fold changes in trisomic and tetrasomic cells compared with parental diploids. **c** Immunoblotting of LC3; GAPDH serves as a loading control. **d** Quantification of the LC3-II/LC3-I ratios upon treatment with Bafilomycin A1 (BafA1) and in control DMSO-treated cells. Each point represents one independent biological experiment, 3–10 experiments were performed in each cell line. **e** Localization of TFEB was assessed by immunofluorescence analysis. White arrows point to the localization of the nucleus in these cells. **f** Quantification of the relative TFEB nuclear localization. The difference in mean fluorescent intensity (MFI) between nuclear and cytoplasmic signals was calculated and plotted relatively to control diploid cells. At least 2000 cells for HCT116 and 1000 cells for RPE1 cell lines were analyzed in each experiment, the means of each experiment are plotted. Each point represents the mean from one independent biological experiment, 4–7 experiments were performed in each cell line. **g** Immunofluorescence images of LC3 and LAMP2 localization in trisomic and diploid cells. White arrows indicate double-positive puncta. **h**, **i** Quantification of LC3- and LAMP2-positive puncta per cell. Number of analyzed cells: *n* = 30 for LC3, *n* = 20 for LAMP2 in each cell line. Unpaired *t*-test was used for statistical analysis, unless otherwise specified. Individual measurements, mean values, *p*-values, and standard deviations are shown in the plots, statistical evaluation is summarized in Supplementary table 4, and source data is available in Supplementary Data 5. Scale bar 10 µm. Mann–Whitney statistical analysis was applied for data in **f**.

Previously, we established isogenic series of human cell lines derived from HCT116 or RPE1 that carry an extra copy of one or more chromosomes^11,38^. The engineered cell lines were named according to their origin (H: HCT116; R: RPE1), the type of chromosome gain (tr: trisomic; te: tetrasomic), and the transferred chromosome (3, 5, 7, 8, 12, 18, or 21), followed by a clone number (e.g., Htr3_11 is a trisomy of chromosome 3 in HCT116, clone 11, Supplementary table 1). Proteome and transcriptome analysis revealed a general upregulation of a wide range of autophagy and lysosomal factors in these cell lines^11,21^. Detailed examination of the transcriptome and proteome data showed that specifically the targets of the transcription factor TFEB, such as LC3 (MAP1LC3B), SQSTM1, VPS18, and WIPI1, as well as cathepsins D, B and A, and other lysosome-specific proteases were upregulated in the analyzed cell lines (Fig. 1a, b and Supplementary Data 1). This upregulation of autophagy and lysosome was observed in different trisomic cells, regardless of their specific karyotype.

Immediately after chromosome missegregation, and thus as part of the acute response to aneuploidy, TFEB also translocates to the nucleus and activates expression of its downstream targets. However, in these settings, autophagy and lysosomal degradation appear to be impaired^39^. To assess autophagic flux in constitutive trisomic cells, we determined the accumulation of lipidated LC3-II, a marker of autophagosome formation, upon addition of the vacuolar H^+^ -ATPase inhibitor bafilomycin A1, and compared it with that of control cells. This analysis showed that autophagic flux was generally unaffected in aneuploid cells, with LC3-II increasing to a similar level or more after bafilomycin treatment than in the parental diploid control (Fig. 1c, d). Additionally, cathepsin D levels were increased, and its activity was not diminished (Supplementary Fig. 1a–c). Importantly, the LC3-II/LC3-I ratio was increased in trisomic cell lines also under normal culture conditions (Supplementary Fig. 1d, e).

TFEB activity is regulated by nucleocytoplasmic transport, and indeed, we observed statistically significant enrichment of TFEB in the nucleus in trisomic and tetrasomic cells compared with the parental control by immunofluorescence (IF) imaging (Fig. 1e, f). TFE3, another factor from the MiT/TFEB family of autophagy and lysosomal gene regulators, was similarly enriched, but mainly in HCT116-derived trisomic cells, likely representing physiological differences between HCT116 and RPE1 cell lines (Supplementary Fig. 1f, g). These data indicate that autophagy is activated in response to chromosome gain, independently of the identity of an extra chromosome. For subsequent analysis of the mechanisms of autophagy activation, we selected five different trisomies, each with a different extra chromosome.

By IF, we additionally confirmed the accumulation of LC3-positive foci (marker of autophagosomes, Fig. 1g, h) in aneuploid cells. Similarly, increased accumulation of lysosomes in aneuploid cells compared with diploids was observed by IF of LAMP2 (marker of lysosomes) and by staining with LysoTracker (Fig. 1g, i and Supplementary Fig. 1h, i). We also used the doubly tagged mRFP–GFP–LC3 to visualize autophagic flux^40^. Because only the mRFP signal is resistant to the acidic pH in lysosomes, this experiment allows us to estimate the ratio between autophagosomes (yellow signal) and autolysosomes (red signal). This analysis confirmed accumulation of autolysosomes in aneuploid cell lines (Supplementary Fig. 1j, k). Taken together, the gain of a chromosome causes increased nuclear localization of MiT/TFEB transcription factors and increased expression of their target genes encoding regulators of autophagy and lysosomal functions; this, in turn, allows chronically aneuploid cells to maintain constitutively elevated autophagy.

### Constitutive nuclear TFEB localization in trisomic cells is mTORC1-independent

TFEB localization is regulated by mTORC1, and reduced activity of this kinase (e.g., during starvation) leads to nuclear accumulation of TFEB^33^. Analysis of the activity of the known upstream mTORC1 regulators AKT1 and AMPK revealed no consistent p-AKT1–S473 changes and unchanged p-AMPK-T172 levels in aneuploid cells compared with diploid parental cells (Supplementary Fig. 2a–d). To investigate mTORC1 activity in trisomic cells, we immunoblotted the cell lysates with an antibody against p-P70S6K-T389, a direct phosphorylation target of mTORC1. We found significantly increased phosphorylation in three out of five trisomic cell lines, confirming that the mTORC1 activity was not uniformly reduced in trisomic cells (Supplementary Fig. 1d and Supplementary Fig. 2a, e). TFEB localization and autophagic flux may also be affected by other cellular stresses, such as oxidative stress or the unfolded protein response (UPR)^34^. Analysis of NRF2 and XBP1 activity, key factors in cellular antioxidant response and UPR, respectively, revealed no significant differences between aneuploid and diploid cell lines (Supplementary Fig. 2f–i).

We also analyzed the changes in autophagy activation in aneuploid and wild-type cells after treatment with the mTORC1 inhibitor Torin 1. Efficient mTORC1 inhibition was confirmed by downregulation of p-P70S6K–T389 and p-ULK1–S575 (Supplementary Fig. 2a, j, k). As expected, inhibition of mTORC1 resulted in an increased ratio of lipidated LC3-II versus nonlipidated LC3-I proteins (Supplementary Fig. 2a, l). Inhibition of mTORC1 should also increase the nuclear localization of TFEB. As expected, nuclear TFEB was enriched in diploid cells after a treatment with Torin 1 to levels comparable to nuclear TFEB enrichment in trisomic cells without any treatment. The highly elevated nuclear TFEB did not increase further in trisomic cells; a slight increase was observed only in Htr21_3, a trisomy cell line with the lowest constitutive TFEB localization (Supplementary Fig. 2m). Our results that mTORC1 inhibition does not further increase nuclear enrichment of TFEB in aneuploid cells suggest that TFEB is nearly maximally activated in cells with extra chromosomes. Based on these results, we conclude that an mTORC1-independent mechanism is responsible for constitutive TFEB nuclear localization and increased gene expression of TFEB targets in human trisomic cells.

### The cGAS–STING–TBK1–IRF3 pathway is activated in constitutively trisomic cells

**Fig. 2:**
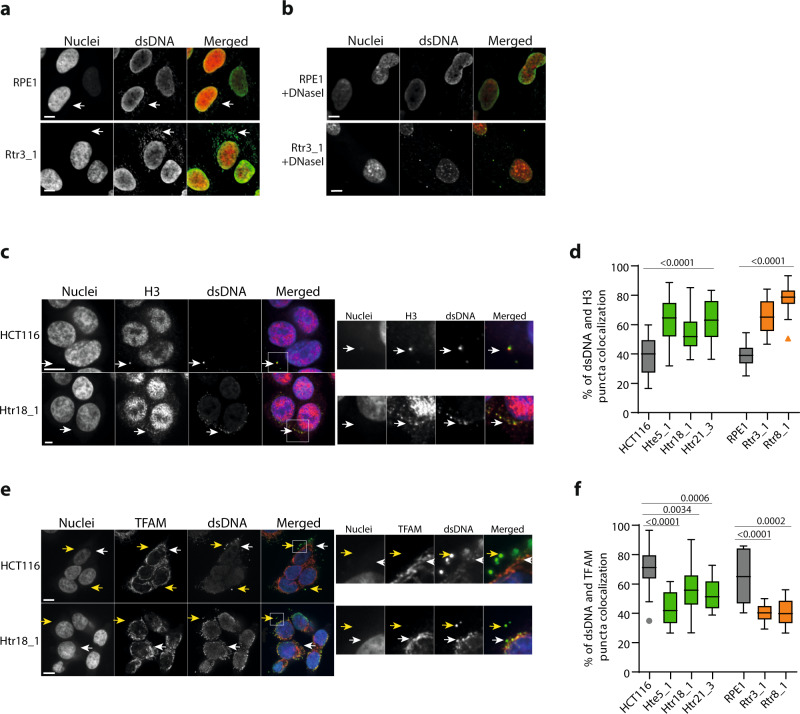
Increased presence of cytoplasmic dsDNA in cells with extra chromosomes. **a** Examples of immunofluorescent images of nuclear and cytoplasmic dsDNA. White arrows point to the dsDNA staining in the cytoplasm. **b** Staining of cytoplasmic dsDNA in cells treated with DNase I. **c** Immunofluorescence images of dsDNA colocalization with H3 (white arrows). **d** Quantification of the percentages of dsDNA puncta copositive for H3. **e** Immunofluorescence of dsDNA with TFAM colocalization (white arrows). Yellow arrows indicate dsDNA puncta negative for TFAM. **f** Quantification of the percentages of puncta positive for both dsDNA and TFAM. Individual measurements, mean values, *p*-values, and standard deviations are shown in the plots, statistical evaluation is summarized in Supplementary table 4, and source data is available in Supplementary Data 5. At least 20 cells were analyzed for the puncta quantification for each cell line. Unpaired *t*-test was used for statistical analysis. Scale bar 10 µm.

Recent evidence has suggested that autophagy can be activated via cGAS–STING signaling independently of mTORC1^37^. We hypothesized that this pathway might contribute to the increased TFEB activity in response to trisomy. This hypothesis is consistent with previous observations that constitutive trisomy activates the type I interferon pathway^11,21,22^. Recently, endogenous DNA damage and replication stress, which is also a hallmark of trisomic cells^3,13,19,20^, has been shown to cause accumulation of cytoplasmic DNA, thereby contributing to activation of the type I interferon pathway^23,25,26,41^. We first analyzed the presence of dsDNA in the cytoplasm using a specific anti-dsDNA antibody, followed by fluorescent imaging. Indeed, trisomic cells showed an increase in cytoplasmic dsDNA compared with control HCT116 and RPE1 cells that was similar to the increase observed in parental diploid cells treated with the DNA damage-inducing agent arabinocytosin (AraC) (Fig. 2a and Supplementary Fig. 3a). DNase I treatment reduced the cytoplasmic dsDNA signal, further supporting the specificity of the antibody (Fig. 2b and Supplementary Fig. 3b). To identify the origin of cytoplasmic dsDNA, we stained the cells with antibodies against histone H3 to label cytosolic fragments of nuclear chromatin^42^. Antibodies against the mitochondrial transcription factor TFAM were used to label the DNA of mitochondrial origin^43^. While ~40% of cytosolic dsDNA colocalized with H3 in diploid cells, this fraction increased to 60–80% in cells with extra chromosomes (Fig. 2c, d and Supplementary Fig 3c). In contrast, almost 80% of cytosolic DNA in diploid cells was of mitochondrial origin, whereas this fraction was significantly reduced in aneuploid cells (Fig. 2e, f and Supplementary Fig 3d, e). Interestingly, cytosolic chromatin is presumably removed via the autophagy pathway, as inhibition of autophagosome–lysosome fusion by treatment with Bafilomycin A1 increased cytosolic dsDNA accumulation in trisomic cells, but not in diploids (Supplementary Fig. 4a, b). These observations are consistent with our hypothesis that DNA leakage from trisomic nuclei into the cytoplasm activates the cGAS–STING pathway.

**Fig. 3:**
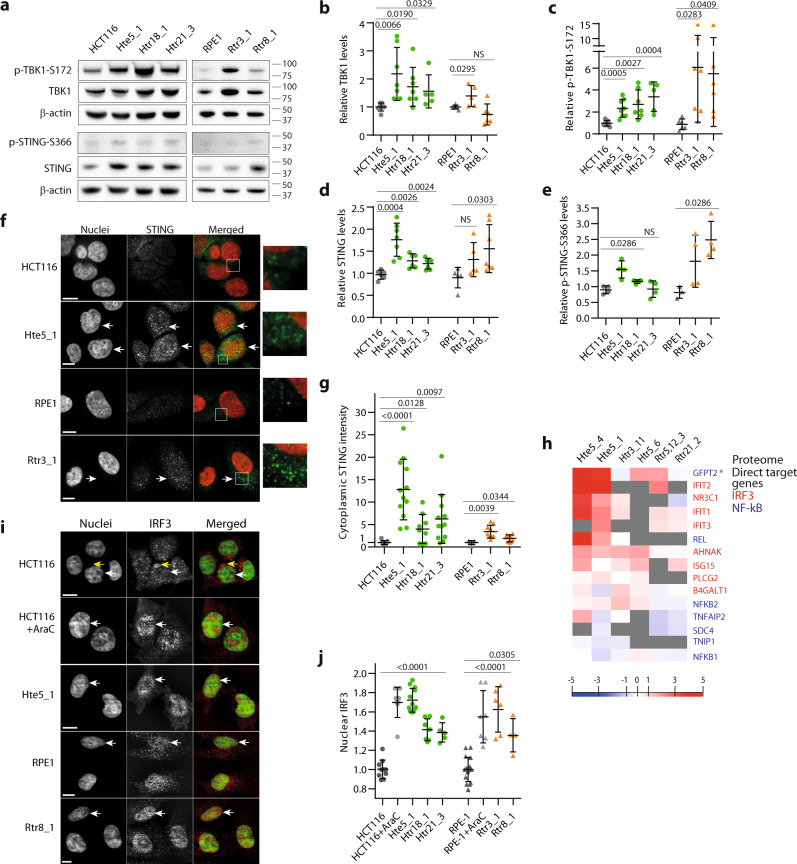
The cGAS–STING–TBK1–IRF3 pathway is constitutively active in trisomic cells. **a** Representative image of immunoblotting of TBK1, p-TBK1–S172, STING, and p-STING–S366. β-actin serves as a loading control. Quantification of TBK1 (**b**), p-TBK1–S172 (**c**), STING (**d**), and p-STING–S366 (**e**) by signal intensity of the protein bands on the gel. The signal was normalized to parental controls. Each point represents the mean from one independent biological experiment, 4–8 experiments were performed in each cell line. **f** Immunofluorescence images of STING localization. White arrows indicate increased clustering in trisomic cells. **g** Quantification of cytoplasmic STING fluorescence intensity. Delta MFI from the total cell and the nucleus was calculated for the cytoplasmic signal. At least 200 cells were analyzed in each independent biological experiment, means of each experiment are shown, and 5–12 experiments were performed in each cell line. **h** Proteome analysis of IRF3 and NF-kB targets. The heat map shows fold changes in trisomic and tetrasomic cells compared with parental diploid cells. *GFPT2 is located on chromosome 5, which may explain its increased abundance in cell lines with trisomy 5. **i** Examples of immunofluorescent images of IRF3 localization. White arrows point to the nuclei. Yellow arrow indicates perinuclear space occupied by IRF3 protein**. j** Quantification of the relative intensity of the nuclear IRF3 signal. Delta MFI data, characterizing IRF3 abundance in the nucleus, were quantified as a difference between nuclear and cytoplasmic MFI for each individual cell. Delta MFI data were plotted after normalization to the parental diploid controls. Diploid cells treated with DNA-damaging agent AraC were used as a positive control. At least 1000 cells were analyzed per each cell line, means of each experiment are shown, and 4–17 independent experiments for each cell line were performed. Unpaired t-test was used for statistical analysis of all the data; in **e** Mann–Whitney test was applied. Individual measurements, mean values, *p*-values, and standard deviations are shown in the plots. Statistical evaluation is summarized in Supplementary table 4, source data is available in Supplementary Data 5. Scale bar 10 µm.

Activation of the cGAS–STING pathway is manifested by phosphorylation and relocalization of pathway members. Indeed, in trisomic cells, we observed increased levels of the downstream kinase TBK1 and p-TBK1–S172, as well as of STING and p-STING–S366 (Fig. 3a–e). Consistent with this observation, IF of STING revealed increased accumulation and clustering of the STING signal in the cytoplasm of trisomic cells (Fig. 3f, g). The cGAS–STING–TBK1 axis triggers expression of the interferon type I factors either via the IRF3 or NF-kB transcriptional factors; transcriptome analysis suggested that specifically targets of IRF3 are overexpressed in trisomic cells (Fig. 3h and Supplementary Data 2). The increased expression of IRF3 targets and ISGs in aneuploid cells was confirmed by qRT-PCR (Supplementary Fig. 5a, b). Activation of IRF3 by chromosome gain was confirmed by its increased nuclear localization in aneuploid cells, which was comparable to IRF3 nuclear accumulation in diploid cells treated with AraC (Fig. 3i, j), or after transformation with plasmid DNA (Supplementary Fig. 5e, f). In contrast, NF-κB targets were not consistently upregulated, as shown by transcriptome analysis and confirmed by qRT-PCR of the canonical NF-kB target IL6 (Fig. 3h and Supplementary Data 3). Activation of the NF-kB pathway is also accompanied by reduced level of its inhibitor IkBα, but its abundance was not reduced in trisomic cell lines (Supplementary Fig. 5c, d). These results indicate that the type I interferon response is triggered via IRF3 activation upon chromosome gain.

Nuclear localization of STAT1, a transcription factor contributing to ISG expression^44^, was also increased in aneuploid cells (Supplementary Fig. 6a, b). Induction of ISG expression in trisomic cell lines was lower compared with induction upon treatment with the synthetic immunostimulant poly-IC or with purified interferon, but comparable to levels observed in parental diploid cells treated with the DNA damage-inducing agent AraC or with the interferon-stimulatory DNA ISD (Supplementary Figs. 5a, b, g and 6c–e). Finally, accumulation of the cGAS product cGAMP was modestly increased in trisomic cells compared with diploid parental cell lines (Supplementary Fig. 6f). Thus, constitutively trisomic cells activate a modest, but persistent inflammatory response via the cGAS–STING–TBK1–IRF3 axis.

**Fig. 4:**
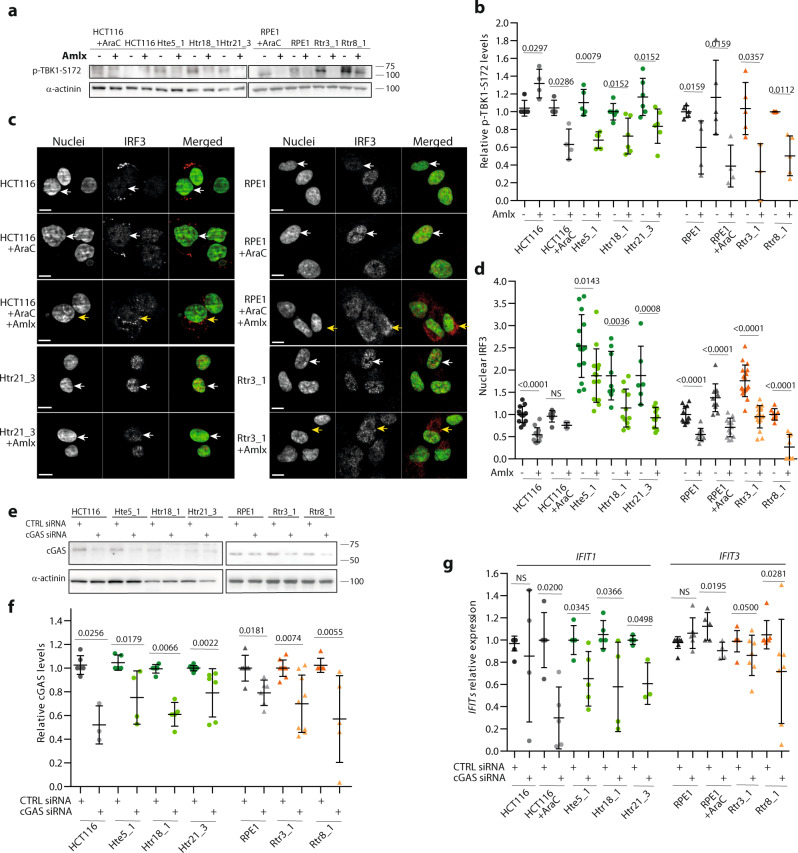
Activation of the innate immune response in constitutive trisomic cells depends on cGAS and TBK1. **a** Representative images of immunoblotting of p-TBK1–S172 inhibition upon treatment with Amlexanox (Amlx) (100 µM in HCT116 and 50 µM in RPE1 cell lines, 4 h of incubation). **b** Quantification of the TBK1 phosphorylation after treatment with Amlx. The data were normalized to the corresponding DMSO controls. In total, 3–6 independent experiments were performed in each cell line. **c** Examples of immunofluorescence images of IRF3 localization after treatment with Amlx. White arrows point to the nuclei and yellow indicates the perinuclear space. Scale bar 10 µm. **d** Quantification of the nuclear IRF3 (delta MFI) after Amlexanox treatment. At least 1500 cells in HCT116 and 500 cells for RPE1-derived cell lines were analyzed in each independent experiment; values normalized to the corresponding control are plotted, 6–17 experiments were performed in each cell line. Unpaired *t*-test was used for statistical analysis. **e** Representative immunoblot of cGAS upon treatment with siRNA. **f** Quantification of the cGAS protein levels upon depletion with cGAS siRNA, 3–9 independent experiments were performed. **g** qRT-PCR data of IRF3 target gene expression changes upon cGAS siRNA. *IFIT1* and *IFIT3* were measured relative to the endogenous control *RPL27* and SPIKE control. All samples were normalized to the corresponding control siRNA. In total, 3–6 independent experiments were performed in each cell line. Mann–Whitney test was applied for statistical analysis, unless otherwise specified. Individual measurements, mean values, *p*-values, and standard deviations are shown in the plots, statistical evaluation is summarized in Supplementary table 4, and source data is available in Supplementary Data 5.

TBK1 kinase is an upstream regulator of the transcription factor IRF3^29^. After treatment with Amlexanox, a selective inhibitor of TBK1 kinase activity, we observed reduced phosphorylation of TBK1 and reduced nuclear localization of IRF3 in all trisomic cell lines (Fig. 4a–d). Additionally, the expression of *OAS3*, *IFIT1*, or *IFIT3* was decreased in the trisomic cell lines after TBK1 inhibition (Supplementary Fig. 7a–c). Thus, active TBK1 is required for the IRF3-dependent gene expression in trisomic cells.

To determine whether the expression of IRF3 targets and ISGs in trisomic cells is mediated by the cGAS–STING pathway, we depleted cGAS with siRNA. The depletion efficiency determined by WB was approximately 50% (Fig. 4e, f). Importantly, this modest cGAS depletion diminished the expression of its targets *IFIT1* or *IFIT3* in constitutive trisomic/tetrasomic cells, and in diploid cells treated with AraC. In contrast, the expression remained largely unchanged in diploid cells upon cGAS depletion (Fig. 4g). We conclude that the gain of a single chromosome causes genotoxic stress that induces type I interferon response via the cGAS–STING–TBK1–IRF3 pathway.

### The cGAS–STING signaling pathway activates autophagy and TFEB-dependent transcription in trisomic cells

**Fig. 5:**
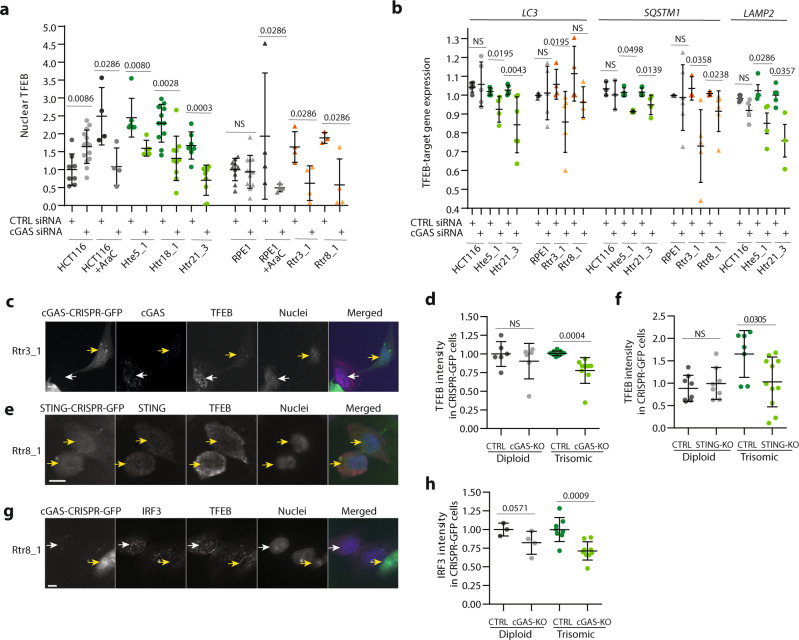
The activation of autophagy in constitutive trisomic cells depends on cGAS. **a** Quantification of TFEB nuclear localization upon cGAS siRNA treatment. The nuclear TFEB was quantified from delta MFI similarly as in Fig. 1f and plotted relative to CTRL siRNA for every cell line. In total, 3–14 independent experiments were performed in each cell line. **b** qRT-PCR data of selected TFEB target genes after cGAS depletion with siRNA. Relative *LC3, SQSTM1*, and *LAMP2* levels were calculated using endogenous controls *RPL27* and SPIKE. The data were plotted after normalization to the corresponding control siRNA sample for each cell line. In total, 3–6 independent experiments were performed for each cell line. **c**, **e**, **g** Examples of immunofluorescence images of TFEB localization upon cGAS or STING knockout with CRISPR/Cas9 nickase. Yellow arrow marks the cells efficiently transfected with the CRISPR–CAS9–GFP RNP that lack either nuclear TFEB (**c**, **e**) or IRF3 (**g**). White arrow marks the untransfected cells with normal nuclear expression of cGAS, IRF3, and TFEB. Scale bar 10 µm. **d**, **f**, **h** Quantification of the MFI of TFEB (**d**, **f**) and IRF3 (**h**) signals in cGAS or STING knockout cells transfected with CRISPR–CAS9–GFP. The data are presented in comparison with cells transfected with control CRISPR–CAS9–GFP. Both HCT116 and RPE1 diploid and all aneuploid cell lines were grouped together. About 4–11 measurements were performed in each sample group. Mann–Whitney test was applied for the statistical analysis of the graphs **a** and **b**, unpaired *t*-test was used for the graphs **d**, **f**, **h**. Individual measurements, mean values, *p* value, and standard deviations are shown in the plots, statistical evaluation is summarized in Supplementary table 4; source data is available in Supplementary Data 5.

**Fig. 6:**
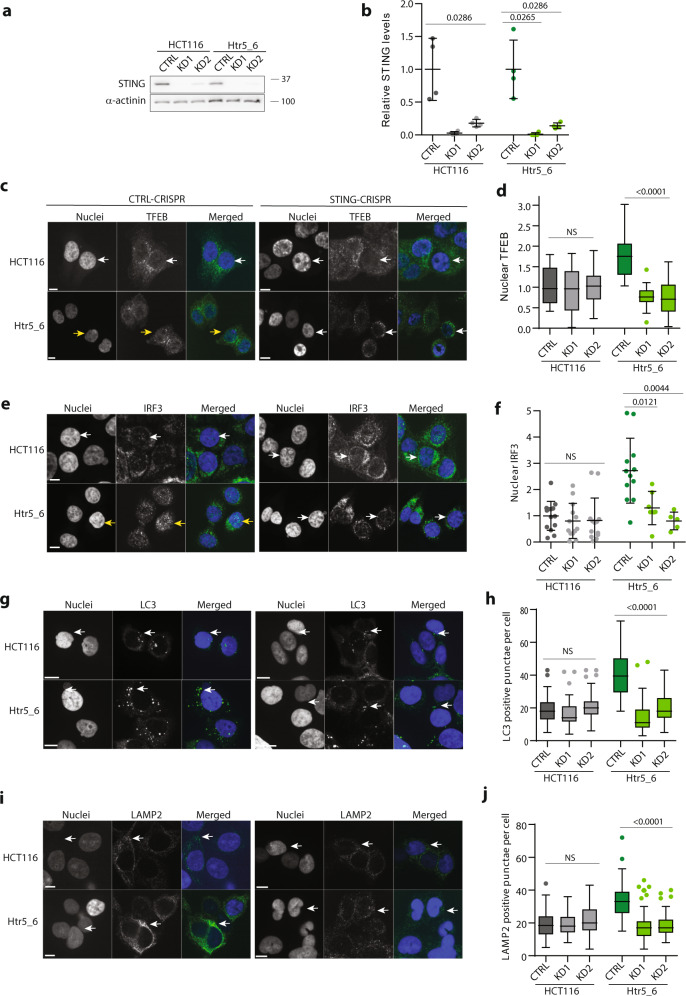
The activation of autophagy in constitutive trisomic cells depends on STING. **a** Immunoblotting of STING protein upon knockdown using the STING–CRISPR–CAS9–KRAB system (two different gRNAs were used, KD1 and KD2). α-actinin was used as a loading control. **b** Quantification of three independent knockdown experiments from a). Four experiments were performed in each cell line. Mann–Whitney test was applied. **c** Immunofluorescence images of TFEB in STING–CRISPR–CAS9–KRAB and CTRL–CRSIPR–CAS9–KRAB-transfected cells. White arrows point to nuclei negative for TFEB and yellow arrows point to the nucleus positive for TFEB. **d** Quantification of nuclear TFEB intensities upon STING knockdown. **e** IRF3 immunofluorescence images in STING-KD cells. White arrows indicate the absence of IRF3 in the nucleus, while yellow arrows indicate IRF3-positive nuclei. In total, 400 cells were analyzed in each cell line, 11–16 independent experiments were performed. **f** Quantification of the IRF3 nuclear intensity in STING-KD cells normalized to the control cells. In total, 400 cells were analyzed in each cell line, means of individual experiments are shown, and 5–14 experiments were performed. **g** LC3 immunofluorescent images with STING-KD. White arrows point to LC3-positive puncta. **h** Quantification of the number of LC3-positive puncta per cell with STING-KD. **i** Immunofluorescence images of LAMP2 in STING–CRISPR–CAS9–KRAB KD cells. White arrows point to LAMP2-positive puncta. **j** Quantification of LAMP2-positive puncta per cells in STING-KD cells. In each experiment, 30–60 cells were analyzed for LC3 and LAMP2 puncta quantification. For all graphs, unpaired two-tailed *t*-test was used for statistical analysis, unless otherwise specified; statistical evaluation is summarized in Supplementary table 4, source data is available in Supplementary Data 5. Individual measurements, mean values, and standard deviations are shown on the plots. Scale bar 10 µm.

We hypothesized that activation of the cGAS–STING pathway is also responsible for autophagy activation in trisomic cells. To test this idea, we depleted cGAS using siRNA and evaluated the markers of autophagy in these cells. For these experiments, we used the same conditions that were sufficient to reduce the innate immune response (Fig. 4e–g). Strikingly, nuclear accumulation of TFEB was reduced to 30–50% in trisomic cell lines upon cGAS depletion, but not in diploids (Fig. 5a). We also detected a modestly reduced expression of the TFEB target genes LC3, SQSTM1, and LAMP2 in trisomic cells, but not in diploid controls (Fig. 5b). Because we achieved only a 50% reduction of the cGAS levels by siRNA, we decided to eliminate cGAS and STING using CRISPR/CAS9 double nickase. By IF, we observed that loss of cGAS and STING significantly reduced the accumulation of nuclear TFEB in trisomic cells, but not in diploid cells (Fig. 5c–f). Loss of cGAS also reduced nuclear IRF3 levels, with diploid cells less affected than trisomic cells (Fig. 5g, h). We additionally used our previously established system of CRISPRi in trisomic cells^45^. For this, we expressed a dCAS9–KRAB CRISPRi vector in diploid control as well as in a cell line trisomic for chromosome 5 (Htr5_6). We then transduced the cell lines with two different guide RNAs (gRNAs) targeting STING. As shown in the WB, both gRNAs strongly reduced STING levels, whereas the control gRNA showed no effect (Fig. 6a, b). Importantly, IF imaging revealed a significant reduction in nuclear accumulation of TFEB and IRF3 after STING depletion in trisomic cells, but not in diploid control cells (Fig. 6c–f). Reduced STING also caused a decrease in cytoplasmic LC3 and LAMP2 signal, but only in trisomic cells (Fig. 6g–j). This confirms our hypothesis that cGAS–STING signaling is required for autophagy activation in response to chromosome gain.

Previously, the cGAS–STING pathway was reported to activate autophagy via mTOR–TBK1^46^. To determine whether nuclear TFEB localization in trisomic cells depends on TBK1, we used Amlexanox to inhibit TBK1 activity. While the LC3-II/LC3-I protein ratio was increased in parental diploids upon Amlexanox treatment, it remained unchanged or was reduced in trisomic cells (Supplementary Fig. 7d, e). Accordingly, nuclear localization of TFEB was not affected by inhibition of TBK1 activity. In fact, nuclear TFEB enrichment further increased after TBK1 inhibition in trisomic cells, but not in diploid cells (Supplementary Fig. 7f, g). In conclusion, cGAS and STING are crucial for both innate immune response and transcriptional activation of autophagy in aneuploid cells, whereas downregulation of TBK1 activity in trisomic cells impairs only the innate immune response.

### Primary cells from trisomy syndromes activate IFN type I response and autophagy via the cGAS–STING signaling pathway

**Fig. 7:**
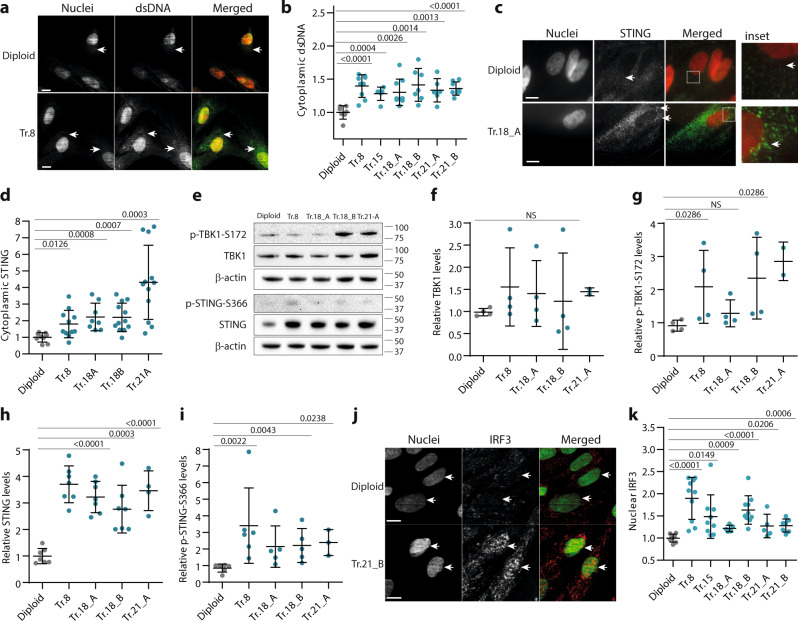
Innate immune response is activated in primary fibroblasts with trisomy. **a** Examples of immunofluorescence images of nuclear and cytoplasmic dsDNA. White arrows point to the dsDNA-positive signal outside of the nucleus. **b** Quantification of the relative intensity of cytoplasmic dsDNA signal. At least 700 cells were analyzed in each experiment, 6–9 experiments were performed in each cell line, and means of individual experiments are plotted. Delta MFI data for the cytoplasmic dsDNA were calculated as the difference between the signals from the total cellular area and the nuclear area; plotted are data normalized to the diploid controls. **c** Immunofluorescence images of STING. White arrows indicate the STING-positive clusters. **d** Quantification of the cytoplasmic STING intensities, calculated similarly as in **b**. In total, 100 cells were analyzed in each experiment, means of each experiment are plotted, and 8–12 experiments were performed for each cell line. **e** Immunoblot showing total and phosphorylated levels of TBK1 and STING proteins and its relative quantification. **f**–**i** In total, 3–7 experiments were performed in each cell line. **j** Localization of IRF3 in primary fibroblasts. White arrows point to the nuclei with a strong nuclear IRF3 signal in trisomic cells. **k** Quantification of the relative IRF3 nuclear localization. At least 1400 cells were analyzed in 5–10 independent experiments, means of each experiment are plotted. Delta MFI data for the nuclear IRF3 were calculated and normalized to the diploid controls. Unpaired two-tailed *t*-test was used for statistical analysis; **f**, **g**, **i** were analyzed with Mann–Whitney test. Individual measurements, mean and *p*-values, and standard deviations are shown in the plots; statistical evaluation is summarized in Supplementary table 4, source data is available in Supplementary Data 5. Scale bar 10 µm.

We next asked whether similar pathways were activated in primary cells from individuals or embryos with trisomy syndromes. Interestingly, cells from individuals with Down syndrome express a specific inflammatory signature that has been attributed to chromosome 21 gene composition, although many of the upregulated genes are not located on this chromosome^47^. We hypothesized that this pattern reflects more a general response to the presence of an extra chromosome. This hypothesis is supported by the fact that the same transcriptional inflammatory signature of Down syndrome patients is also found in our model trisomies, regardless of the identity of the additional chromosome (Supplementary Fig. 8a and Supplementary Data 4). To determine whether the constitutive cGAS–STING-dependent activation of TFEB and IRF3 is general to naturally occurring trisomies, we compared six primary fibroblast cultures from embryonic material trisomic for chromosomes 8, 15, 18, or 21 with diploid primary fibroblasts. By IF, we detected a significant increase in cytoplasmic dsDNA of nuclear origin in all trisomic primary embryonic cells compared with the diploid control (Fig. 7a, b and Supplementary Fig. 8b–g). By WB, we observed increased levels of STING, p-STING, and p-TBK1–S172, as well as increased STING clustering in the cytoplasm in trisomic cells (Fig. 7c–i). Accordingly, nuclear localization of IRF3 was significantly enhanced in all trisomic primary cells compared with the diploid control (Fig. 7j, k and Supplementary Fig. 8h). Finally, by mass spectrometry, we observed an increased cGAMP production in Tr.21 primary fibroblasts (Supplementary Fig. 8i). This suggests that accumulation of cytoplasmic dsDNA in primary trisomic fibroblasts contributes to the chronic innate immune response, similar to what we observed in model trisomic cell lines.

**Fig. 8:**
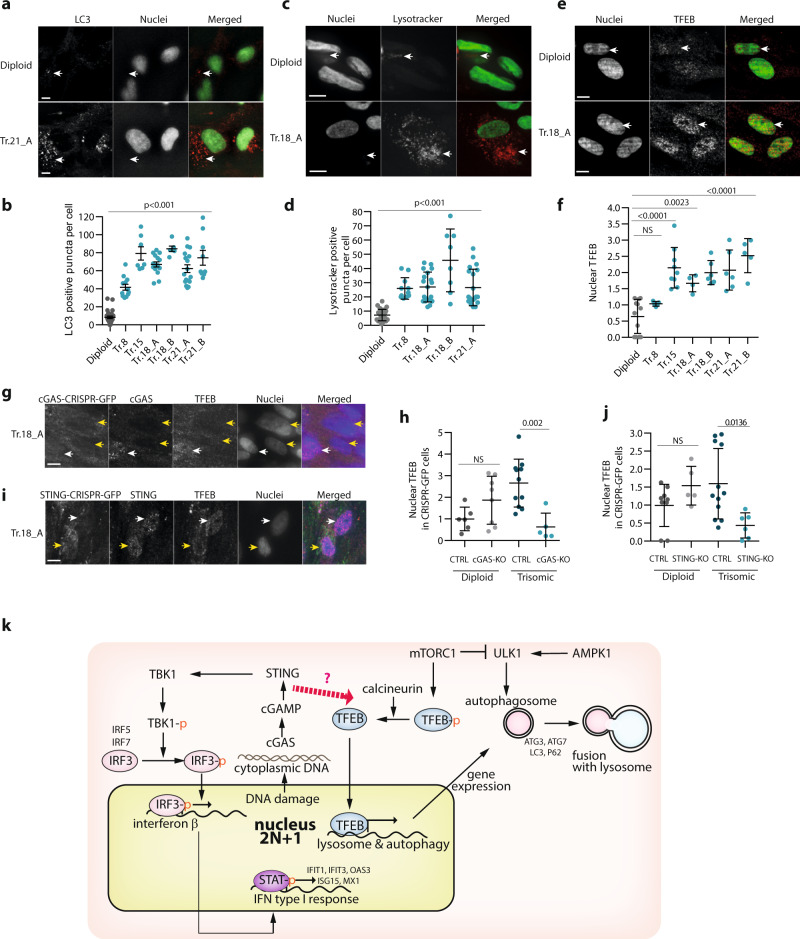
Autophagy is activated in primary fibroblasts with trisomy. **a** Immunofluorescence images of LC3 in trisomic fibroblasts. White arrows indicate LC3-positive puncta. **b** Quantification of the numbers of LC3-positive puncta per cell. In total, 6–25 cells were analyzed in each experiment. **c** Representative images of cells labeled with lysotracker. **d** Quantification of the number of lysotracker-positive puncta per cell. In total, 8–25 cells were analyzed in each experiment. **e** Representative images of TFEB localization in primary fibroblasts. White arrows indicate nuclei positive for the TFEB protein. **f** Quantification of the relative nuclear TFEB signal. Delta MFI data were calculated similarly as in Fig. 1f and plotted after normalization to diploid control cells. At least 1400 cells were analyzed in 5–9 independent experiments, means of each experiment are plotted. **g**, **i** Representative images of TFEB localization in trisomic fibroblasts transfected with GFP-positive cGAS–CRISPR–CAS9 RNP (**g**) or with STING–CRISPR–CAS9 RNP (**i**). Transfected cells (GFP positive) are marked with yellow arrows, untransfected cells with white arrows. **h**, **j** Quantification of nuclear TFEB intensity inhibited upon cGAS (**g**) and STING (**i**) CRISPR–CAS9-KO. Data were normalized to the control transfected with CTRL–CRISPR–CAS9 RNP. In total, 5–12 cells were analyzed in each group. Unpaired *t*-test was used for statistical analysis. Individual measurements, mean and *p*-values, and standard deviations are shown on the plots, statistical evaluation is summarized in Supplementary table 4, and source data is available in Supplementary Data 5. Scale bar 10 µm in all images. **k** Schematics of cGAS–STING involvement in TFEB-mediated activation of autophagy in trisomic cells. DNA damage due to genotoxic stress in trisomic cells leads to accumulation of dsDNA in the cytosol, where it is bound by the cytosolic receptor cGAS. Activated cGAS produces cGAMP -signaling molecule that induces STING clustering and TBK1 activation and, subsequently, IRF3-dependent transcription of its target genes. Additionally, activated STING triggers translocation of TFEB to the nucleus in an mTOR-independent manner. In this context, TFEB induces expression of lysosomal and autophagy factors, which in turn promotes autophagy and lysosomal degradation.

Finally, we asked whether autophagy was increased in these cells. Indeed, the primary fibroblasts showed significantly increased levels of cytoplasmic LC3 puncta, lysosomes, as well as accumulation of nuclear TFEB (Fig. 8a–f and Supplementary Fig. 9a). An increased LC3-II/LC3-I protein ratio was observed in all trisomic primary fibroblasts (Supplementary Fig. 9b, c). This was accompanied by increased relative autophagic flux, as documented by an increased LC3-II/LC3-I protein ratio after treatment with Bafilomycin 1A (Supplementary Fig. 9d, e). Importantly, the increased autophagic flux was independent of mTORC1 activity, as most trisomic cells showed increased p-P70S6K–T389 phosphorylation and no significant changes in p-ULK1–S757 levels (Supplementary Fig. 9b, f, g). To confirm that the cGAS–STING pathway contributes to autophagy activation in primary trisomic fibroblasts, we again employed the CRISRP/CAS9-mediated knockout of STING and cGAS. Indeed, loss of these two factors significantly reduced the accumulation of nuclear TFEB in trisomic primary fibroblasts, similarly as observed in model trisomic cell lines (Fig. 8g–j). Taken together, our data suggest that chromosome gain induces interferon type I response and autophagy via the cGAS–STING–IRF3 signaling pathway.

## Discussion

Model constitutive trisomic cell lines have provided useful insights into the consequences of abnormal chromosome copy numbers^13^. A gain of a single chromosome in somatic mammalian cells triggers a conserved gene expression pattern that reflects the proteotoxic and genotoxic stress in these cells. Correspondingly, trisomic cells suffer from impaired protein folding and increased proteasomal and autophagic activity, as well as from an accumulation of DNA damage and impaired replication. Human trisomic cells also show increased expression of several factors that are involved in interferon type I signaling^21,22^. Activation of both the inflammatory response and autophagy has been previously recognized in trisomic cells, but the upstream triggers have never been identified. Here, we show that these three phenotypes—increased DNA damage, autophagy activation, and interferon response—are intimately linked in aneuploid cells. We demonstrate that increased DNA damage leads to accumulation of cytoplasmic dsDNA in trisomic cells that, in turn, activates the cGAS–STING-mediated innate immune response. This further activates the TBK1/IRF3-dependent expression of interferon-stimulated genes, as well as the TFEB-dependent expression of lysosomal and autophagosomal factors (Fig. 8k).

Previous data revealed an increased expression of autophagosomal and lysosomal factors and increased levels of autophagosomes and lysosomes accompanied by a reliance on phagolysosome activity in somatic trisomic mammalian cells^11,17^. The presence of extra chromosomes leads to the production of excessive proteins that may overwhelm the capacity of lysosomal degradation, as was observed in cells acutely missegregating chromosomes^39^. We propose that cells with extra chromosomes adapt to the altered protein homeostasis and increased requirement for autophagy by constitutive nuclear localization of TFEB, which augments the expression of the required autophagy and lysosomal factors. Strikingly, the constitutive nuclear localization of TFEB is independent of mTOR in trisomic cells. Several mTORC1-independent mechanisms of TFEB activation were recently described, including through the activation of AKT kinase or via PERK, which is activated as a part of the ER stress-induced unfolded protein response^48,49^. While these pathways may contribute to TFEB activation in individual trisomic cell lines, our data suggest that none of them serves as a universal trigger of nuclear TFEB localization and transcriptional activation of autophagy in our model system as well as in primary embryonic fibroblasts. Instead, we propose that nuclear TFEB localization depends on the cGAS–STING pathway.

Can trisomy per se activate the cGAS–STING pathway? Here, we demonstrate that trisomic cells contain increased levels of cytoplasmic dsDNA, a well-known activator of the cGAS–STING pathway.

Work from several laboratories recently revealed that increased DNA damage can lead to elevated levels of cytoplasmic dsDNA, which is recognized by the cytosolic receptor cGAS^23,25,41^. Gain of a chromosome was shown to cause genotoxic stress due to abnormal replication^3,19,20^, and we propose that this may be the reason for the accumulation of dsDNA in the cytosol of trisomic cells. The DNA may also originate from damaged mitochondria that are often found in trisomic cells^50^; however, our data suggest rather a nuclear origin of the cytosolic DNA. Micronuclei arising from missegregation of chromosomes were previously reported to activate the cGAS–STING pathway and, subsequently, the NF-κB-mediated transcription^51–53^; however, the used model trisomic cell lines do not missegregate chromosomes at a significantly higher rate than the parental diploids^20^. Thus, chromosomally unstable cells that continuously missegregate chromosomes, thereby producing micronuclei, may activate cGAS–STING via a different mechanism than constitutive trisomies with low rates of chromosomal instability.

It should be noted that the abundance of the cGAS protein and the cGAS–STING activity varies in different cell lines. Indeed, there is some discrepancy regarding the detection of cGAS expression in HCT116 and RPE1 cell lines, where some laboratories have found no cGAS protein^54^, while others did (e.g., refs. ^45,55–57^). We show that there is a functional cGAS–STING pathway in HCT116 and RPE1 cell lines that becomes chronically activated in constitutively aneuploid cells. This finding is also supported by data we obtained in primary fibroblasts from trisomic embryos.

Importantly, cytoplasmic dsDNA that accumulates in trisomic cells activates the cGAS–STING pathway, as documented by increased cGAMP production, followed by increased TBK1 kinase activity. Our data demonstrate that TBK1, via IRF3 and STAT1, is responsible for the elevated expression of type I interferon response and interferon-stimulated genes in trisomic cells. The observed activation of the innate immune response in multiple different trisomic cells was consistent, significant, and independent of the identity of the extra chromosome, although markedly lower than upon interferon stimulation or poly-IC transfection. This is consistent with previous observations of a moderate increase in ISG expression in response to genotoxic conditions^52,58,59^. Collectively, our data show that a gain of even a single chromosome induces modest, but chronic activation of the innate immune pathway accompanied by ISG expression. In future experiments, it should be determined whether and how the expression of ISGs affects the survival and proliferation of trisomic cells. Moreover, while we analyzed several different trisomic cell lines, it remains to be determined whether this phenotype is universal, or whether it can be influenced by the identity of the extra chromosome.

Recently, an evolutionary conserved role of cGAS–STING signaling in autophagy activation was proposed^37^. Here, we demonstrate that the nuclear localization of TFEB in model trisomic cells at least partly depends on cGAS activity. cGAS–STING can activate TFEB via TBK1 and mTORC1, as has been proposed using chronic immune activation in a Trex1^−/−^ mouse model^60^, but also via unknown mechanisms independently of TBK1^37^, suggesting that the involvement of TBK1 in autophagy regulation depends on the context. We show that inhibition of TBK1 did not influence autophagy in trisomic cells, nor did it reduce the nuclear localization of TFEB. The exact mechanism of TFEB regulation upon cGAS–STING pathway activation in trisomic cells will be the subject of future studies.

We made the initial observations in our model human cell lines engineered to carry one or two extra copies of individual chromosomes, but, notably, the observed phenotypes also hold true for primary embryonic fibroblasts from trisomic embryos. By testing four different trisomies from six embryos of different sex and origin, we demonstrate that these cells accumulate cytoplasmic dsDNA and activate the innate immune response as well as autophagy in an mTORC1-independent manner. Individuals with Down syndrome are often predisposed to autoimmune disorders, such as insulin-dependent diabetes mellitus, celiac disease, and others^61^. Recently, it was proposed that DS individuals exhibit dysregulated interferon signaling due to the chr. 21-specific overexpression of immune factors. Yet, transcriptional analysis of differentially regulated genes in cells from patients with trisomy 21 showed that the top 13 upregulated factors are IFN-related factors, which are mostly not encoded on chromosome 21^47,62^. We present here evidence that the upregulated interferon response in trisomic cells is independent of the identity of the extra chromosome, as we observed the same response in cells trisomic for chromosomes 8, 15, 18, or 21. We propose that the upregulation instead arises as a direct consequence of constitutive innate immune pathway activation in trisomic cells that leads to low-grade inflammation.

Our data, together with previously published findings, suggest the following model (Fig. 8k). Cells with any additional chromosome suffer from a chronically imbalanced proteome that causes well- documented aneuploidy-associated stresses^7,13^. This negatively impacts DNA replication and repair, which leads to accumulation of cytoplasmic dsDNA and subsequent activation of the cGAS–STING pathway. We propose that the cGAS–STING signaling not only activates the type I interferon response, but also executes a transcriptional program to upregulate autophagy and lysosomal biogenesis independently of mTORC1 signaling, thus connecting genetic instability of trisomic cells to autophagy activation. Our findings provide a rationale for why aneuploid cells are recognized by the immune system and removed from tissues^2,3^. These cellular changes occur independently of the identity of the extra chromosome and occur also in primary embryonic fibroblasts with trisomy syndromes. Importantly, the observed activation of innate immunity by chromosome gain provides a new insight into possible causes of chronic low-grade inflammation that is frequently observed in cancer and in trisomy syndromes.

## Methods

### Cells used in the study and culture conditions

RPE1 hTERT (referred to as RPE1) and RPE1 hTERT H2B-GFP were a kind gift from Stephen Taylor (University of Manchester, UK). HCT116 H2B-GFP was generated by lipofection (FugeneHD, Roche) of HCT116 (ATCC No. CCL-247) with pBOS–H2B–GFP (BD Pharmingen) according to the manufacturer’s protocols^63^. Trisomic and tetrasomic cell lines were generated by microcell-mediated chromosome transfer as previously described^11^. The cell line Hte5_1 was kindly provided by Minoru Koi, Baylor University Medical Centre, Dallas, TX, USA. All cell lines were maintained at 37 °C with 5% CO2 atmosphere in Dulbecco’s Modified Eagle Medium (DMEM) containing 10% fetal bovine serum (FBS), 100 U penicillin, and 100 U streptomycin. All cell lines tested negative for mycoplasma contamination. The human primary embryonic fibroblasts were purchased from the cell repository of Coriell Institute for Medical Research, NJ 08103, USA. Cells were propagated in similar culture conditions for up to three passages and DMEM was supplemented with 15% of FBS. All experiments were performed at subconfluent conditions. The full list of cells used in the study is presented in Supplementary table 1.

### siRNA, ISD, and DNA transfection

The siRNA transfection was performed according to the manufacturer’s protocol. In total, 2 × 10^5^ cells per well were seeded in DMEM media with supplements in a six-well dish and incubated overnight; media was replaced with DMEM without antibiotics 4 h ahead of the transfection. First, 20 pmol siRNA (cGAS siRNA sc-95512, CTRL siRNA sc-36869, Santa Cruz) was diluted in 100 µL of transfection medium (SC-36868, Santa Cruz) and 6 µL of transfection reagent (SC-29528, Santa Cruz) were diluted to 100 µL with transfection medium. Then, siRNA and transfection reagent solution were gently mixed and incubated at room temperature for 30 min. Next, the entire 200-µL siRNA/transfection solution was added, dropwise, on top of cells. Cells were then left to incubate for 16 h. The next day, the media was exchanged for fully supplemented media, and the cells were incubated for 24 h before collection for RT-qPCR, Western blot, or immunofluorescence. Double- stranded ISD DNA oligo (10 μg) and plasmid DNA were transfected with a similar strategy and collected after 4 and 6 h for qPCR analysis.

### Drug treatment

For Amlexanox (4857, Tocris) experiments, 2.5 × 10^5^ RPE1 cells or 1 × 10^6^ HCT116 cells were seeded into 6-cm dishes and incubated overnight. The next day, 100 µM or 50 µM concentrations were used for RPE1 and HCT116 cell lines, respectively. For western blot, cells were incubated with Amlexanox for 4 h, which was sufficient to decrease the levels of p-TBK1–S172. For qRT-PCR experiments, cells were incubated overnight. For the positive control, AraC (C6645, Sigma) was added at 50 µM to the control cells and incubated overnight. To inhibit mTOR complex activity, Torin 1 (4247, Tocris) was applied overnight at a concentration of 2 µM. To measure the autophagic flux, the lysosome inhibitor Bafilomycin A1 (1334, Tocris) was used for 4 h at 100 nM.

### CRISPR/CAS9 cGAS and STING depletion

cGAS and STING Double Nickase Plasmid (sc-403354-NIC, sc-403148-NIC, Santa Cruz Biotechnology) was used to remove the target protein. Control Double Nickase Plasmid was used in parallel in the same experimental conditions (sc-437281, Santa Cruz Biotechnology). The transfection procedure was performed according to the manufacturer’s protocol available online, using UltraCruz Transfection Reagent (sc-395739, Santa Cruz Biotechnology), Plasmid Transfection Medium (sc-108062, Santa Cruz Biotechnology). The transfected cells were visualized with GFP encoded by the same plasmids.

An alternative approach to depleted STING was to use HCT116 and Htr5_6 cell lines that stably express dCAS9–KRAB. Using viral transduction, we introduced two variable guide RNAs for successful STING knockdown, in a similar strategy as we described before^45^.

### Immunoblotting

To prepare the whole-cell lysate, cells were lysed with RIPA buffer and sonicated. After spinning down, the supernatant was used for protein concentration measurements with the Bradford protein assay. To prepare samples for SDS-PAGE, the whole-cell lysate was diluted to 1 µg/µL with Lämmli solution and water, and the samples were boiled for 5 min at 95 °C. Gels were loaded with 10 µg of sample and run for 15 min at 100 V, followed by 210 V for 35 min. Precision Plus 50 Protein All Blue Standard was used as a marker. The proteins were transferred onto a nitrocellulose blotting membrane via semidry transfer using Bjerrum Schafer–Nielson transfer buffer and the Trans-Blot® Turbo™ (BioRad Laboratories, Hercules, USA). Next, the membranes were blocked for 30 min with 5% milk solution, and primary antibodies were added and followed by incubation at 4 °C overnight. The next day, secondary antibodies were added followed by incubation for 1 h at room temperature. To visualize the signal on the membranes, a horseradish peroxidase solution (ECL) was used and imaged with the Azure c500 system (Azure Biosystems, Dublin, USA). ImageJ software was then used to quantify each protein band intensity, which was then normalized to its respective loading control. Protein-loading amount was controlled for either using Ponceau staining or α-actinin intensity. Information regarding the used antibodies can be found in Supplementary table 2. All immunoblotting experiments were performed in at least three biological replicates.

### Immunofluorescence

HCT116 and RPE1 cells and their aneuploid derivatives were seeded to a 96-well plate (HCT116: 10^4^ cells per well, RPE1: 10^3^ cells per well) and incubated at 37 °C and 5% CO_2_ on the day before experiments. For cell fixation, a 3% formaldehyde solution was used for 15 min at room temperature. Next, cells were permeabilized with 0.1% Triton for 20 min at room temperature. Then, cells were preblocked with 3% bovine serum for 30 min at room temperature. Primary antibodies were diluted in blocking solution (Supplementary table 2) and incubated overnight at 4 °C. The next day, cells were washed and secondary antibodies were added in a concentration of 1 mg per ml followed by incubation for 1 h in the dark at room temperature. SYTOX® green (Invitrogen) or DAPI (Invitrogen) were used to localize the nucleus. For cytoplasmic staining, the HCS Cell Mask (H327 12 Component, 701618, Invitrogen) was applied. The cells were covered with SlowFade Gold Antifade (Invitrogen). To localize mitochondria, MitoTracker^TM^ Red CMXRos (M7512, Invitrogen) was used (100 nM for 45-h incubation at 37 °C). For lysosome localization, we also used LysoTracker^TM^ Red DND-99 (L7528, Thermofisher Scientific) according to the manufacturer’s protocol. Information regarding the used antibodies can be found in Supplementary table 2.

### Autophagy activity assay

HCT116 and RPE1 diploid and aneuploid cells were seeded 1 day before transfection to achieve 40% confluency at the transfection day. The ptfLC3 plasmid (mRFG-GFP-LC3, Addgene) was transfected using Lipofectamine 2000 according to the manufacturer’s protocol. Two days after transfection, cells were fixed with ice-cold methanol for 10 min and SlowFade Gold Antifade reagent with DAPI. mRFP-positive (red puncta) autolysosomes and GFP/mRFP-positive (yellow puncta) autophagosomes were visualized with ×60 objective and counted to estimate the autophagy flux.

### Microscopy

Spinning-disk confocal laser microscopy was performed using a fully automated Zeiss inverted microscope (AxioObserver Z1) equipped with a MS-2000 stage (Applied Scientific Instrumentation, Eugene, OR), the CSU-X1 spinning-disk confocal head (Yokogawa), and LaserStack Launch with selectable laser lines (Intelligent Imaging Innovations, Denver, CO). Image acquisition was performed using a CoolSnap HQ camera (Roper Scientific) and a 20x-air, 40x-air, or 63x-oil objective (Plan Neofluar × 40/0.75, Plan Neofluar ×20/0.75) under the control of the SlideBook 6 × 64 program (SlideBook Software, Intelligent Imaging Innovations, Denver, CO, USA).

### Image analysis

CellProfiler, a cell image analysis software (https://cellprofiler.org/), was used to quantify microscopy images. A mask of the nucleus of each cell using nuclear staining was taken and applied to an image of specific staining to quantify the nuclear signal. To quantify the cytoplasmic signal intensity, the mask area was extended around the nucleus, without counting the nucleus. The difference in values for nuclear and cytoplasmic signals of specific staining was used to determine increased or decreased presence of proteins in the nucleus. To collect the full signal from the cytoplasm, the cell mask was used and the nuclear signal was subtracted as described above. Three independent biological replicates were performed for each experiment, at least 300 cells were scored in each experiment. In the CRISPR/Cas9 experiment with CRISPR–GFP double-nickase experiment, all imaged cells were scored.

### Quantitative real-time PCR

To isolate RNA, Qiagen instructions for RNeasy Mini Kit and On-column Dnase Digestion (Qiagen) were followed. cDNA was made with the iScript^TM^ Advanced cDNA Synthesis Kit (1725037, BioRad) using 2 µg of RNA. SPIKE (RS25SI, TATAA Biocenter AB) was used as an exogenous reference to control for technical variability. RPL27 primers were used as an endogenous reference to control for RNA quantity and quality in each sample. qPCR was performed in a 96-well plate, where each sample was loaded in triplicate, according to the SYBR Master Mix manufacturer’s instructions. The results were examined with the BioRad CFX Manager. Primer sequences are in Supplementary table 3.

### Transcriptome and proteome analysis

Genome-wide proteome and transcriptome expression profiling of HCT116- and RPE1-derived aneuploid cell lines was previously performed^11,21^. As previously described, bioinformatics analysis of the proteomic and transcriptomic data was performed using Perseus (1.6.2.3) as part of the MaxQuant Software Package. For comparison of each aneuploid cell line with the corresponding control cell line, gene expression fold change ratios were calculated. We used the same datasets to analyze autophagy- and immune response-specific gene expression. To address the changes in autophagy- and lysosomal-specific gene expression, we used the published gene set including TFEB target genes^64^. To investigate the type I IFN response, the subgroups of “IRF3 direct targets” and “NF-κB-dependent genes” were used to show gene enrichment from specific transcription factors^65,66^.

### Quantification of cGAMP

ELISA kit (501700, Cayman chemicals) was used to quantify the amount of 2′3′-cGAMP in cell lysates. The procedure was performed according to the manufacturer’s protocol. Additionally, cGAMP levels in primary fibroblasts were analyzed by means of LC–MS/MS with a 4000 QTrap (AB Sciex, Darmstadt, 3 Germany) and a Dionex UHPLC UltiMate 3000 (Thermo Fisher) or Prominence HPLC (Shimadzu, 4 Germany) similarly as was previously described for nucleotide quantification.

### Oxidative stress measurements

Cells were incubated with CellROX™ Deep Red Reagent (Invitrogen C10422) according to the manufacturer’s instructions and analyzed on a BD FACSCalibur flow cytometer. As a positive control for reactive oxygen species generation, control cells were incubated with 100 µM Menadione (M5625, Merck) for 90 min.

### Cathepsin D activity measurements

Cathepsin D activity assay kit (ab65302, Abcam) was used to quantify the activity of Cathepsin D in cell lysates. The procedure was performed according to the manufacturer’s protocol.

### Statistics and reproducibility

Statistical analyses were performed from at least three independent experiments. The number of the performed independent measurements is specified in the respective figure legends and illustrated in Supplementary data 5 and 6. GraphPad Prism software was used for the statistic tests. Statistical analysis was performed using unpaired *t*-test or Mann–Whitney test as indicated in the corresponding figure legends. Values are shown as the mean±sd of multiple independent experiments. For imaging analysis, the number of analyzed cells by CellProfiler software is stated in the figure legends for each cell line.

To identify significantly upregulated genes, a modified T-test adjusted for multiple testing (FDR = 0.05, S0 = 0.1, Perseus) was used to evaluate the normalized log2 mRNA and protein intensities obtained from trisomic cell lines.

### Reporting summary

Further information on research design is available in the Nature Research Reporting Summary linked to this article.

## Supplementary information

Supplementary Information

Description of Additional Supplementary Files

Supplementary Data 1

Supplementary Data 2

Supplementary Data 3

Supplementary Data 4

Supplementary Data 5

Supplementary Data 6

Reporting Summary

## Data Availability

All data generated or analyzed during this study are included in this published article (and its Supplementary information files). Source data are available in Supplementary Data 5 and 6.
