# Supplementary material for: Genotoxic stress in constitutive trisomies induces autophagy and the innate immune response via the cGAS-STING pathway
Source: Commun Biol. 2021 Jul 2;4:831. doi: 10.1038/s42003-021-02278-9 (PMC8253785; doi:10.1038/s42003-021-02278-9)
Supplement: Supplementary file 1 — Supplementary Information [file 42003_2021_2278_MOESM1_ESM.pdf]

Supplementary Information

Genotoxic stress in constitutive trisomies induces autophagy and the innate immune response via the cGAS-STING pathway

Maria Krivega[1], Clara M. Stiefel[1], Sahar Karbassi[1], Line L. Andersen[3], Narendra K. Chunduri[1], Neysan Donnelly[2], Andreas Pichlmair[3,4], Zuzana Storchova[1,*]

1 Molecular Genetics, University of Kaiserslautern, Paul-Ehrlich Strasse 24, 67663 Kaiserslautern, Germany
2 Max Planck Institute of Biochemistry, Am Klopferspitz 18, 82152 Martinsried, Germany
3 Institute of Virology, TU Munich, Schneckenburgerstrasse 8, 81675 München, Germany
4 German Center for Infection Research (DZIF), Munich partner site, Germany

Corresponding author: Zuzana Storchova, storchova@bio.uni-kl.de, ORCID: 0000-0003-2376-7047

Supplementary figure 1: Characterisation of autophagy in trisomic cells.
Supplementary figure 2: Regulation of autophagy in trisomic cells.
Supplementary figure 3: dsDNA presence in the cytoplasm of trisomic cells.
Supplementary figure 4: dsDNA accumulation in cytoplasm upon autophagy inhibition in trisomic cells.
Supplementary figure 5: Activation of the innate immune pathway in trisomic cells.
Supplementary figure 6: Transcriptional activation of the innate immune pathway in trisomic cells
Supplementary figure 7: Activation of the innate immune response, but not autophagy, depends on TBK1 in constitutive trisomic cells.
Supplementary figure 8: Innate immune response in trisomic human primary fibroblast.
Supplementary figure 9: Autophagy activity in primary human fibroblasts with trisomy.

Supplementary table 1: Cell lines used in the study
Supplementary table 2: List of used antibodies
Supplementary table 3: Primers used for qRT-PCR
Supplementary table 4 Summary of the statistical evaluation for each figure.

Supplementary Data 1: Expression of TFEB-dependent factors in aneuploid cells
Supplementary Data 2: Expression of IRF3 and NFkB-dependent factors in aneuploid cells
Supplementary Data 3: Expression of NFkB targets evaluated by quantitative rtPCR
Supplementary Data 4: Expression of inflammation markers in aneuploid cells
Supplementary Data 5: Source data for all main figures
Supplementary Data 6: Source data for all supplementary figures

Supplementary table 1. Cell lines used in the study**.**

HCT116-derived cell lines					
	**Name**	**Chr.**	**H2B-GFP**	**Type of aneuploidy**	**Comments**
	HCT116	-	-		AATC
	HCT116 H2B-GFP	-	+		Kuffer et al, 2013
1	Htr3_11	3	+	Trisomy	Passerini et al., 2016
2	Hte5_1	5	-	Tetrasomy	From Minoru Koi (Stingele et al, 2012)
3	Htr5_6	5	+	Trisomy	Stingele et al, 2012
4	Hte5_4	5	+	Tetrasomy	Stingele et al, 2012
5	Htr8_5	8	+	Trisomy	Donnelly et al, 2014
6	Htr13_2	13	-	Trisomy	Domingues et al, 2017
7	Htr18_1	18	-	Trisomy	Domingues et al, 2017
8	Htr21_3	21	-	Trisomy	Domingues et al, 2017
**RPE1-derived cell lines**					
	**Name**	**Chr.**	**H2B-GFP**	**Type of aneuploidy**	**Comments**
	RPE1	-	-		Kind gift from Steven Taylor
	RPE1 H2B-GFP	-	+		Kind gift from Steven Taylor
1	Rtr3_1	3	-	Trisomy	Stingele et al, 2012
2	Rtr5,12_3	5, 12	+	Trisomy	Stiengele et al, 2012
3	Rtr7_1	7	+	Trisomy	Duerrbaum et al, 2018
4	Rtr8_1	8	-	Trisomy	Kneissig et al, 2019
5	Rtr21_2	21	+	Trisomy	Stingele et al, 2012

Human primary embryonic fibroblasts				
	**Name**	**Chr.**	**Type of aneuploidy**	**Reference number**
	Diploid ctrl_A			AG04392
	Diploid ctrl_B			AG21708
1	Tr.8	8	Trisomy	GM00496
2	Tr.15	15	Trisomy	GM07189
3	Tr.18_A	18	Trisomy	GM03538
4	Tr.18_B	18	Trisomy	GM00734
5	Tr.21_A	21	Trisomy	GM02058
6	Tr.21_B	21	Trisomy	GM04616

Supplementary table 2 List of used antibodies

Antibody	Conditions used		Reference	Company
	WB	IF		
AKT	1:1000	NA	9272	Santa Cruz Biotechnology
p-Akt (Ser473)	1:1000	NA	4060	Cell Signaling Technology
AMPKα (D5A2)	1:1000	NA	5831	Cell Signaling Technology
p-AMPKα-Thr172	1:1000	NA	2531	Cell Signaling Technology
α-actinin (H-2)	1:1000	NA	17829	Santa Cruz Biotechnology
β-actin-HRP	1:2500	NA	sc-47778	Santa Cruz Biotechnology
cGAS (D1D3G)	1:1000	1:100	15102	Cell Signaling Technology
dsDNA	NA	1:50	58749	Santa Cruz Biotechnology
GAPDH	1:1000	NA	14C10	Cell Signaling Technology
Histone H3	NA	1:100	9715	Cell Signaling Technology
HRP Mouse	1:5000	NA	HAF007	R&D Systems
HRP Rabbit	1:5000	NA	HAF008	R&D Systems
H3	1:500	NA	ab12079	Abcam
IκBα (L35A5)	1:1000	NA	48144S	Cell Signaling Technology
IRF3 (D83B9)	NA	1:50	4302	Cell Signaling Technology
LAMP2	NA	2 µg/mL	ab13534	Abcam
LC3B (EPR18709)	1:3000	2 µg/mL	192890	Abcam
mTOR (EPR390(N))	1:500	NA	ab134903	Abcam
NRF2 (D1Z9C) XP	1:1000	1:100	12721	Cell Signaling Technology
P70S6K	1:1000	NA	2708	Cell Signaling Technology
p-P70S6 (Thr389)	1:500	NA	9205	Cell Signaling Technology
STAT1	NA	1:100	N9172	Cell Signaling Technology
STING (D2P2F)	1:1000	1:50	13647S	Cell Signaling Technology
p-STING (S366) (D7C3S)	1:1000	NA	19781S	Cell Signaling Technology
TBK1 (108A429)	1 µg/mL	NA	Ab12116	Abcam
p-TBK1 (Ser172)(D52C2)	1:500	NA	54834	Cell Signaling Technology
TFAM	NA	2 µg/mL	ab176558	Abcam
TFE3	NA	2 µg/mL	HPA023881	Sigma (Atlas antibodies)
TFEB	NA	5 µg/mL	MAB9170	R&D systems
p-ULK1 (Ser757) (D7O6U)	1:1000	NA	14202	Cell Signaling

Supplementary table 3. Primers used for qRT-PCR

**Target gene**	**Primer sequence (5' → 3')**	**Reference**
hIFIT1 F	TACCTGGACAAGGTGGAGAA	Rubina Baglio et al, 2016
hIFIT1 R	GTGAGGACATGTTGGCTAGA	Rubina Baglio et al, 2016
hIFIT3 F	CTTACGGCAAGCTGAAGAGT	Rubina Baglio et al, 2016
hIFIT3 R	AATTGCCAGTCCAGAGGAGA	Rubina Baglio et al, 2016
hOAS3 F	CCCTGGTCTGAGACTCACGTTT	Ma et al, 2008
hOAS3 R	GACTTGTGGCTTGGGTTTGAC	Ma et al, 2008
hRPL27 F	ATCGCCAAGAGATCAAAGATAA	Donnelly et al, 2014
hRPL27 R	TCTGAAGACATCCTTATTGACG	Donnelly et al, 2014
SPIKE	Proprietary to TATAA Biocenter AB	
LC3 F	GAGAAGCAGCTTCCTGTTCTGG	Jiang et al., 2011
LC3 R	GTGTCCGTTCACCAACAGGAAG	Jiang et al., 2011
SQSTM1 F	ATTGAGTCCCTCTCCCAGAT	Park et al., 2019
SQSTM1 R	CGCTCCGATGTCAT AGTTCTT	Park et al., 2019

Supplementary table 4 Summary of the statistical evaluation for each figure.

Figure 1				
ANOVA, p values		t test, p value		
Fig. 1f IF nuclear TFEB				
HCT116 vs Hte5_1, Htr13_2, Htr18_1, Htr21_3	<0.0001	HCT116 vs	Hte5_1	<0.0001
			H13_2	0.0004
			Htr18_3	0.0003
			Htr21_1	0.0001
RPE1 vs Rtr3_1, Rtr5_3, Rtr8_1, Rtr7_1, Rtr21_2	<0.0001	RPE1 vs	Rtr3_1	<0.0001
			Rtr5_3	<0.0001
			Rtr8_1	<0.0001
			Rtr7_1	0.0101
			Rtr21_2	<0.0001
Fig. 1h IF LC3+puncta				
HCT116 vs Hte5_1, Htr18_1, Htr21_3	<0.0001	HCT116 vs	Hte5_1	<0.0001
			Htr18_3	<0.0001
			Htr21_1	<0.0001
RPE1 vs Rtr3_1, Rtr8_1	<0.0001	RPE1 vs	Rtr3_1	<0.0001
			Rtr8_1	<0.0001
Fig. 1i IF LAMP2+ puncta				
HCT116 vs Hte5_1, Htr18_1, Htr21_3	<0.0001	HCT116 vs	Hte5_1	<0.0001
			Htr18_3	<0.0001
			Htr21_1	<0.0001
RPE1 vs Rtr3_1, Rtr8_1	<0.0001	RPE1 vs	Rtr3_1	0.0088
			Rtr8_1	<0.0001

Figure 2				
ANOVA, p values		t test, p value		
Fig. 2d IF dsDNA and H3 + puncta, %				
HCT116 vs Hte5_1, Htr18_1, Htr21_3	<0.0001	HCT116 vs	Hte5_1	<0.0001
			Htr18_3	<0.0001
			Htr21_1	<0.0001
RPE1 vs Rtr3_1, Rtr8_1	<0.0001	RPE1 vs	Rtr3_1	<0.0001
			Rtr8_1	<0.0001
Fig. 2 IF dsDNA and TFAM + puncta, %				
HCT116 vs Hte5_1, Htr18_1, Htr21_3	<0.0001	HCT116 vs	Hte5_1	<0.0001
			Htr18_3	0.0034
			Htr21_1	0.0006
RPE1 vs Rtr3_1, Rtr8_1	0.0004	RPE1 vs	Rtr3_1	<0.0001
			Rtr8_1	0.0002

Figure 3				
ANOVA, p values		t test, p value		
Fig. 3b WB TBK1				
HCT116 vs Hte5_1, Htr18_1, Htr21_3	0.0252	HCT116 vs	Hte5_1	0.0066
			Htr18_3	0.0190
			Htr21_1	0.0329
RPE1 vs Rtr3_1, Rtr8_1	0.0102	RPE1 vs	Rtr3_1	0.0295
			Rtr8_1	NS
Fig. 3c WB p-TBK1				
HCT116 vs Hte5_1, Htr18_1, Htr21_3	0.0011	HCT116 vs	Hte5_1	0.0005
			Htr18_3	0.0027
			Htr21_1	0.0004
RPE1 vs Rtr3_1, Rtr8_1	NS	RPE1 vs	Rtr3_1	0.0283
			Rtr8_1	0.0409
Fig. 3d WB STING				
HCT116 vs Hte5_1, Htr18_1, Htr21_3	<0.0001	HCT116 vs	Hte5_1	0.0004
			Htr18_3	0.0026
			Htr21_1	0.0024
RPE1 vs Rtr3_1, Rtr8_1	NS	RPE1 vs	Rtr3_1	NS
			Rtr8_1	0.0303
Fig. 3e WB p-STING				
HCT116 vs Hte5_1, Htr18_1, Htr21_3	0.0022	HCT116 vs	Hte5_1	0.0286
			Htr18_3	0.0286
			Htr21_1	NS
RPE1 vs Rtr3_1, Rtr8_1	0.0254	RPE1 vs	Rtr3_1	0.0286
			Rtr8_1	0.0286
Fig. 3g IF cyt. STING				
HCT116 vs Hte5_1, Htr18_1, Htr21_3	<0.0001	HCT116 vs	Hte5_1	<0.0001
			Htr18_3	0.0128
			Htr21_1	0.0097
RPE1 vs Rtr3_1, Rtr8_1	0.0018	RPE1 vs	Rtr3_1	0.0039
			Rtr8_1	0.0344
Fig. 3j IF IRF3 nucl.				
HCT116 vs HCT116+Arac, Hte5_1, Htr18_1, Htr21_3	<0.0001	HCT116 vs	HCT116+AraC	<0.0001
			Hte5_1	<0.0001
			Htr18_3	<0.0001
			Htr21_1	<0.0001
RPE1 vs RPE1+AraC, Rtr3_1, Rtr8_1	<0.0001	RPE1 vs	RPE1+AraC	<0.0001
			Rtr3_1	<0.0001
			Rtr8_1	0.0305

Figure 4		
	Welch's test, p values	t test, p value
Fig. 4g, qRT-PCR *IFITs*, ctrl siRNA vs cGAS siRNA		
HCT116	NS	NS
HCT116+AraC	0.0040	0.0200
Hte5_1	0.0311	0.0345
Htr18_1	NS	0.0366
Htr21_3	NS	0.0498
RPE1	NS	NS
RPE1+AraC	0.0171	0.0195
Rtr3_1	NS	0.0500
Rtr8_1	0.1226	0.0281

Figure 5		
	Welch's test, p values	t test, p value
Fig. 5a IF TFEB nucl., ctrl siRNA vs cGAS siRNA		
HCT116	0.0057	0.0086
HCT116+AraC	0.0322	0.0286
Hte5_1	0.0125	0.0080
Htr18_1	0.0012	0.0028
Htr21_3	0.0003	0.0003
RPE1	NS	NS
RPE1+AraC	NS	0.0286
Rtr3_1	0.0257	0.0286
Rtr8_1	0.0390	0.0286
Fig. 5d – cGAS-CRISPR-KO vs CTRL-CRISPR – TFEB nucl. IF		
Diploid	NS	NS
Trisomic	0.0044	0.0004
Fig. 5f – STING-CRISPR-KO vs CTRL-CRISPR – TFEB nucl IF		
Diploid	NS	NS
Trisomic	0.0318	0.0305
Fig. 5h – cGAS-CRISPR-KO vs CTRL-CRISPR – IRF3 nucl. IF		
Diploid	NS	NS
Trisomic	0.0013	0.0009

Figure 6				
ANOVA, p values		t test, p value		
Fig. 6d IF TFEB nucl.				
HCT116 CTRL vs KD1, KD2	NS	HCT116 CTRL vs	KD1	NS
			KD2	NS
Htr5-6 CTRL vs KD1, KD2	<0.0001	Htr5_6 vs	KD1	<0.0001
			KD2	<0.0001
Fig. 6f IF IRF3 nucl.				
HCT116 CTRL vs KD1, KD2	NS	HCT116 CTRL vs	KD1	NS
			KD2	NS
Htr5-6 CTRL vs KD1, KD2	0.0016	Htr5_6 vs	KD1	0.0121
			KD2	0.0044
Fig. 6h IF LC3+puncta				
HCT116 CTRL vs KD1, KD2	0.0168	HCT116 CTRL vs	KD1	NS
			KD2	NS
Htr5-6 CTRL vs KD1, KD2	<0.0001	Htr5_6 vs	KD1	<0.0001
			KD2	<0.0001
Fig. 6i IF LAMP2+puncta				
HCT116 CTRL vs KD1, KD2	NS	HCT116 CTRL vs	KD1	NS
			KD2	NS
Htr5-6 CTRL vs KD1, KD2	<0.0001	Htr5_6 vs	KD1	<0.0001
			KD2	<0.0001

Figure 7				
ANOVA, p values		t test, p value		
Fig. 7b IF cyt. dsDNA				
Diploid vs Tr.8, Tr.15, Tr.18_A, Tr.18_B, Tr.21_A, Tr.21_B	0.0005	Diploid vs	Tr.8	<0.0001
			Tr.15	0.0004
			Tr.18_A	0.0026
			Tr.18_B	0.0014
			Tr.21_A	0.0013
			Tr.21_B	<0.0001
Fig. 7d IF cyt. STING				
Diploid vs Tr.8, Tr.18_A, Tr.18_B, Tr.21_A	<0.0001	Diploid vs	Tr.8	0.0126
			Tr.18_A	0.0008
			Tr.18_B	0.0007
			Tr.21_A	0.0003
Fig. 7f WB TBK1				
Diploid vs Tr.8, Tr.18_A, Tr.18_B, Tr.21_A	NS	Diploid vs	Tr.8	NS
			Tr.18_A	NS
			Tr.18_B	NS
			Tr.21_A	NS
Fig. 7g WB p-TBK1				
Diploid vs Tr.8, Tr.18_A, Tr.18_B, Tr.21_A	NS	Diploid vs	Tr.8	0.0286
			Tr.18_A	NS
			Tr.18_B	0.0286
Fig. 7h WB STING				
Diploid vs Tr.8, Tr.18_A, Tr.18_B, Tr.21_A	<0.0001	Diploid vs	Tr.8	<0.0001
			Tr.18_A	<0.0001
			Tr.18_B	0.0003
			Tr.21_A	<0.0001
Fig. 7i WB p-STING				
Diploid vs Tr.8, Tr.18_A, Tr.18_B, Tr.21_A	NS	Diploid vs	Tr.8	0.0022
			Tr.18_A	0.0043
			Tr.18_B	0.0043
			Tr.21_A	0.0238
Fig. 7k IF IRF3 nucl.				
Diploid vs Tr.8, Tr.15, Tr.18_A, Tr.18_B, Tr.21_A, Tr.21_B	<0.0001	Diploid vs	Tr.8	<0.0001
			Tr.15	0.0149
			Tr.18_A	0.0009
			Tr.18_B	<0.0001
			Tr.21_A	0.0206
			Tr.21_B	0.0006

Figure 8				
ANOVA, p values		t test, p value		
Fig. 8b IF LC3+puncta				
Diploid vs Tr.8, Tr.15, Tr.18_A, Tr.18_B, Tr.21_A, Tr.21_B	<0.0001	Diploid vs	Tr.8	<0.0001
			Tr.15	<0.0001
			Tr.18_A	<0.0001
			Tr.18_B	<0.0001
			Tr.21_A	<0.0001
			Tr.21_B	<0.0001
Fig. 8d Lysotracker+ puncta				
Diploid vs Tr.8, Tr.18_A, Tr.18_B, Tr.21_A	<0.0001	Diploid vs	Tr.8	<0.0001
			Tr.18_A	<0.0001
			Tr.18_B	<0.0001
			Tr.21_A	<0.0001
Fig. 8f IF TFEB nucl.				
Diploid vs Tr.8, Tr.15, Tr.18_A, Tr.18_B, Tr.21_A, Tr.21_B	<0.0001	Diploid vs	Tr.8	NS
			Tr.15	<0.0001
			Tr.18_A	0.0023
			Tr.18_B	<0.0001
			Tr.21_A	<0.0001
			Tr.21_B	<0.0001

Figure 8		
	Welch's test, p values	t test, p value
Fig. 8h – cGAS-CRISPR-KO vs CTRL-CRISPR – TFEB nucl. IF		
Diploid	NS	NS
Trisomic	0.0006	0.0002
Fig. 8j – STING-CRISPR-KO vs CTRL-CRISPR – TFEB nucl. IF		
Diploid	NS	NS
Trisomic	0.0023	0.0136

Supplementary figure 5				
ANOVA, p values		t test, p value		
Suppl. Fig. 5a qRT-PCR *OAS3*				
HCT116 vs HCT116+Arac, Hte5_1, Htr18_1, Htr21_3	<0.0001	HCT116 vs	HCT116+AraC	0.0040
			Hte5_1	0.0159
			Htr18_1	0.0028
			Htr21_3	0.0033
RPE1 vs RPE1+AraC, Rtr3_1, Rtr8_1	0.0157	RPE1 vs	RPE1+AraC	0.0179
			Rtr3_1	0.0436
			Rtr8_1	NS
Suppl.Fig.5b qRT-PCR *IFITs*				
IFIT1 - HCT116 vs HCT116+Arac, Hte5_1, Htr18_1, Htr21_3	NS	HCT116 vs	HCT116+AraC	0.0286
			Hte5_1	0.0286
			Htr18_1	0.0159
			Htr21_3	0.0095
IFIT3 - RPE1 vs RPE1+AraC, Rtr3_1, Rtr8_1	NS	RPE1 vs	RPE1+AraC	0.0131
			Rtr3_1	0.0179
			Rtr8_1	0.0179

Supplementary figure 5		
	Welch's test, p values	t test, p value
Suppl. Fig. 5f IF IRF3 nucl., transfected vs. nontransfected		
HCT116	<0.0001	<0.0001
RPE1	<0.0001	<0.0001

Supplementary figure 6				
ANOVA, p values		t test, p value		
Suppl. Fig. 6c qRT-PCR *OAS3*				
HCT116 vs HCT116+Arac, Hte5_14, Hte5_1	0.0001	HCT116 vs	HCT116+Arac	0.0040
			Hte5_14	0.0319
			Hte5_1	0.0319
RPE1 vs RPE1+AraC, Rtr21_2	NS	RPE1 vs	RPE1+AraC	0.0179
			Rtr21_2	0.0151
Suppl.Fig.6d qRT-PCR *IFIT1*				
HCT116 vs HCT116+Arac, Hte5_14, Hte5_1	0.0289	HCT116 vs	HCT116+Arac	0.0286
			Hte5_14	0.0571
			Hte5_1	0.0571
RPE1 vs RPE1+AraC, Rtr21_2	0.0048	RPE1 vs	RPE1+AraC	0.0057
			Rtr21_2	0.0057
Suppl. Fig. 6e qRT-PCR *IFIT3*				
HCT116 vs HCT116+Arac, Hte5_14, Hte5_1	0.0030	HCT116 vs	HCT116+Arac	0.0319
			Hte5_14	0.0319
			Hte5_1	0.0319
RPE1 vs RPE1+AraC, Rtr21_2	<0.0001	RPE1 vs	RPE1+AraC	0.0131
			Rtr21_2	0.0265

Supplementary figure 8				
ANOVA, p values		t test, p value		
Fig. 8e IF dsDNA and H3 + puncta, %				
Diploid vs Tr.8, Tr.18_A, Tr.18_B, Tr.21_A	<0.0001	Diploid vs	Tr.8	<0.0001
			Tr.18_A	<0.0001
			Tr.18_B	<0.0001
			Tr.21_A	<0.0001
Fig. 8g IF dsDNA and TFAM + puncta, %				
Diploid vs Tr.8, Tr.18_A, Tr.18_B, Tr.21_A	<0.0001	Diploid vs	Tr.8	<0.0001
			Tr.18_A	<0.0001
			Tr.18_B	<0.0001
			Tr.21_A	<0.0001

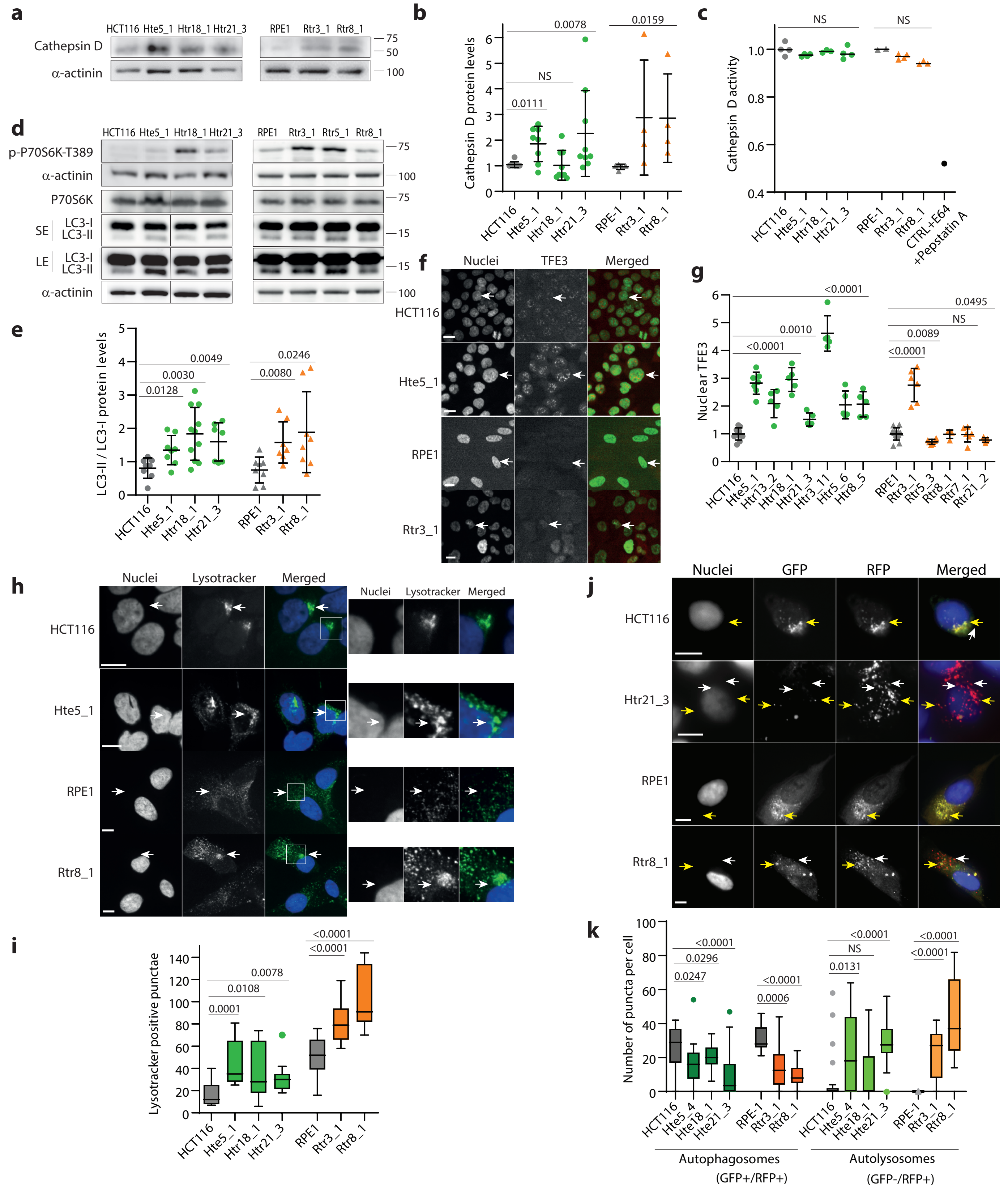

Supplementary Figure 1

Characterisation of autophagy in trisomic cells. Immunoblotting of Cathepsin D protein (a) and its quantification relative to the loading control α-actinin (b), n=4-9. c) Activity of Cathepsin D, n=2-4. Mann-Whitney test was applied for the statistical analysis of the data on plots b) and c). d) Immunoblotting of P70S6K and its phosphorylated form, and of LC3. α-actinin serves as a loading control. SE - short exposure time, LE – long exposure time. e) Quantification of the LC3-II/LC3-I ratio, n=7-11. Imaging and quantification of nuclear TFE3 (f, g). The immunofluorescence data were analysed as in Fig. 1e to quantify the delta MFI. In average at least 1000 cells for HCT116- and 500 cells for RPE1-derived cell lines were analyzed, means of individual experiments are shown, n=4-12. Lysosomes were visualized by lysotracker (h) and positive puncta were manually quantified from at least 15 cells in each cell line (i), n=13-15. Imaging (j) and quantification (k) of yellow GFP+/mRFP+ autophagosomes (yellow arrows) and mRFP+ autolysosomes (white arrows) in cells transfected with LC3-GFP-mRFP construct. At least 20 cells per each condition were analyzed for puncta quantification, number of measurements for statistics n=10-22. Unpaired t test was applied for statistical analysis, unless other is specified. Individual measurements, mean values and standard deviation are shown. Scale bar in all images 10 μm . Statistical evaluation are in Supplementary table 4, source da

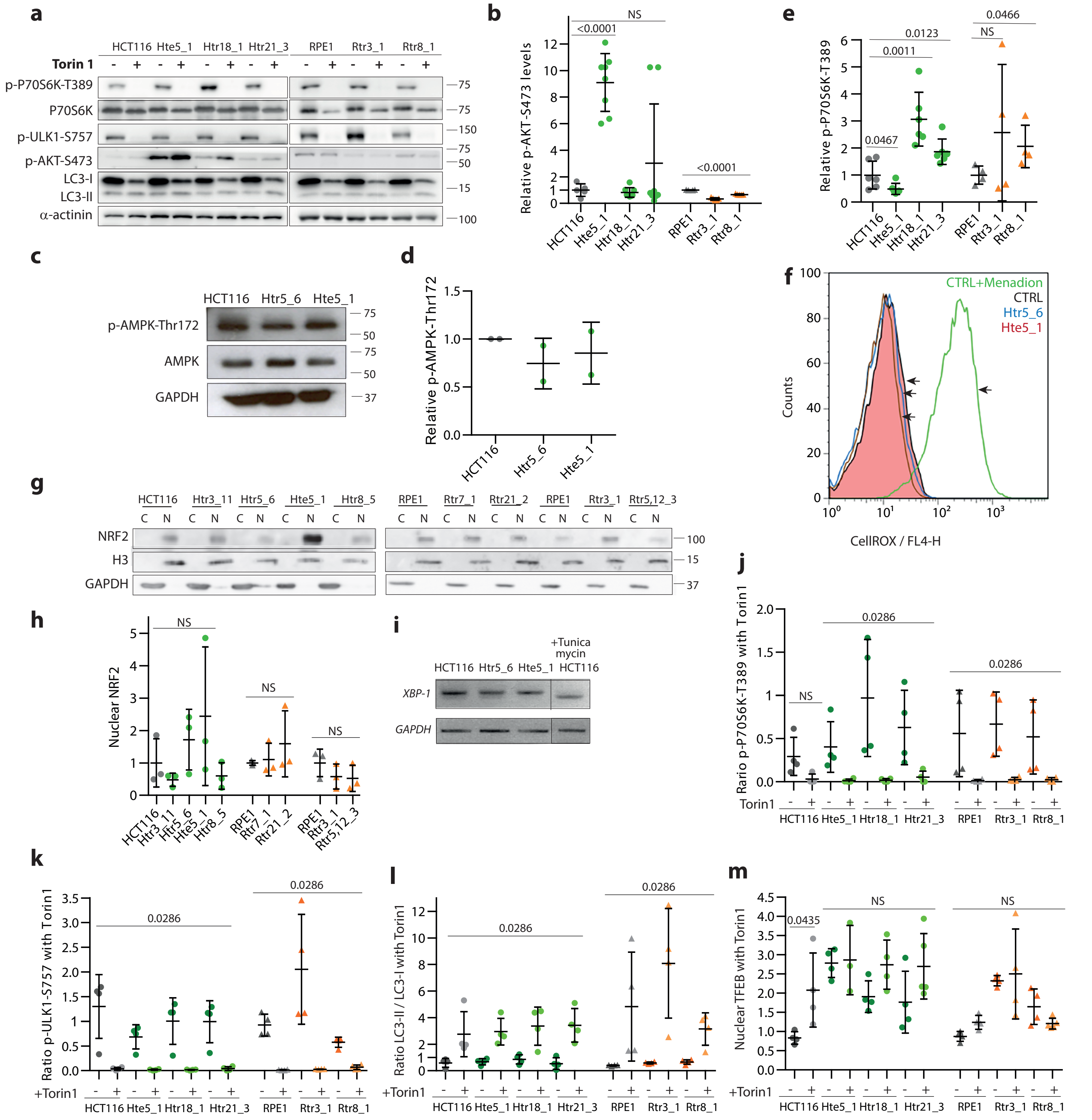

Supplementary Figure 2

Regulation of autophagy in trisomic cells. a) Immunoblotting of mTOR downstream phosphorylation targets p-P70S6K-T389, p-AKT-S473, p-ULK1-S575, and LC3 upon treatment with Torin 1 (2 μM, overnight incubation). α-actinin serves as a loading control. Quantification of the relative phosphorylated p-Akt1-S473 (b, n=4-8) and p-P70S6K-T389 (e, n=4-6). For graphs b) and e) unpaired t test was used for statistical analysis. c) Immunoblotting and d) quantification of the relative phospho-AMPK-Thr172 levels. f) Flow cytometry evaluation of the CellROX signal. g) Immunoblotting and h) quantification of nuclear NRF2 levels in fractionated cell lysates, n=3. i) Electrophoresis of the XBP1 cleavage, HCT116 treated with Tunicamycin was used as a control for unfolded protein response. Quantification of the phosphorylation of p-P70S6K-T389 (j, n=4) and p-ULK1-S757 (k, n=4), inhibited upon treatment with Torin 1. Data were normalized to the corresponding DMSO control for every cell line. l) Quantification of LC3-II/LC3-I ratio upregulation upon inhibition of mTORC1 with Torin 1 (2 μM, overnight incubation), n=4. m) Quantification of nuclear TFEB localization upon inhibition of mTORC1 with Torin 1 (2 μM, overnight incubation), n=3-5. The immunofluorescence data were analysed as in Fig. 1c in order to quantify the delta MFI relative to control. The sample analysis was performed relative to corresponding DMSO control. Mann-Whitney test was applied for all graphs, unless other wisespecified. Individual measurements, mean values and standard deviation are shown. Statistical evaluation and source data are summarized in Supplementary table 4 and Supplementary Data 6, respectively.

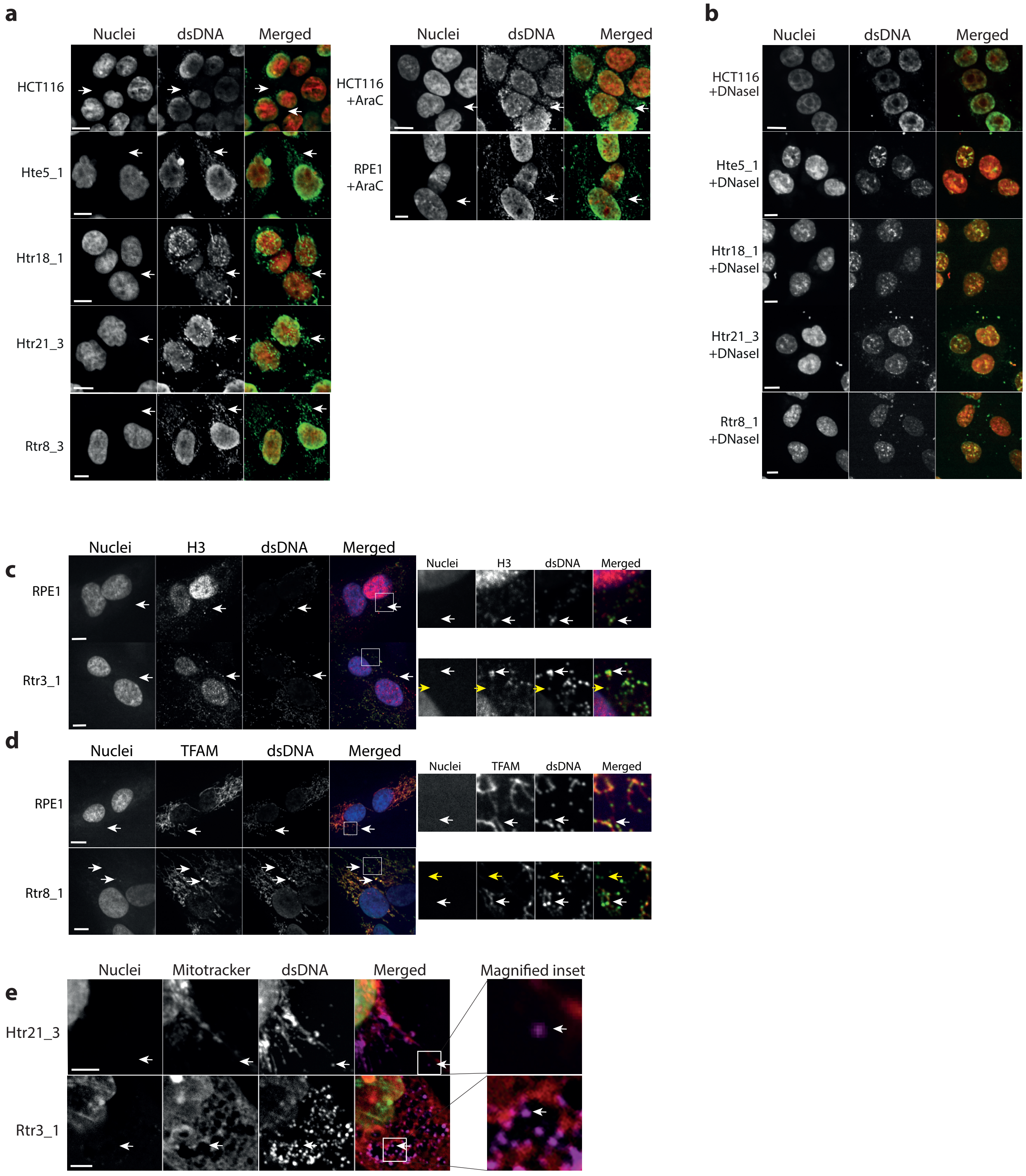

Supplementary Figure 3

dsDNA presence in the cytoplasm of trisomic cells. Microscopy images of cytoplasmic dsDNA (white arrows) in trisomic and control cells treated with AraC, as a positive control, (a) and DNase I as a negative control (b). c) Images of cytoplasmic dsDNA and mitotracker. White points on colocalization of dsDNA and mitotracker in cytoplasm of the cell. Imaging of cytoplasmic dsDNA colocalized with genomic H3 (d) and mitochondrial TFAM (e). White arrows indicate dsDNA puncta that colocalize with H3 (d) or TFAM (e) and yellow arrows that didn't. Scale bar in all images 10 µm.

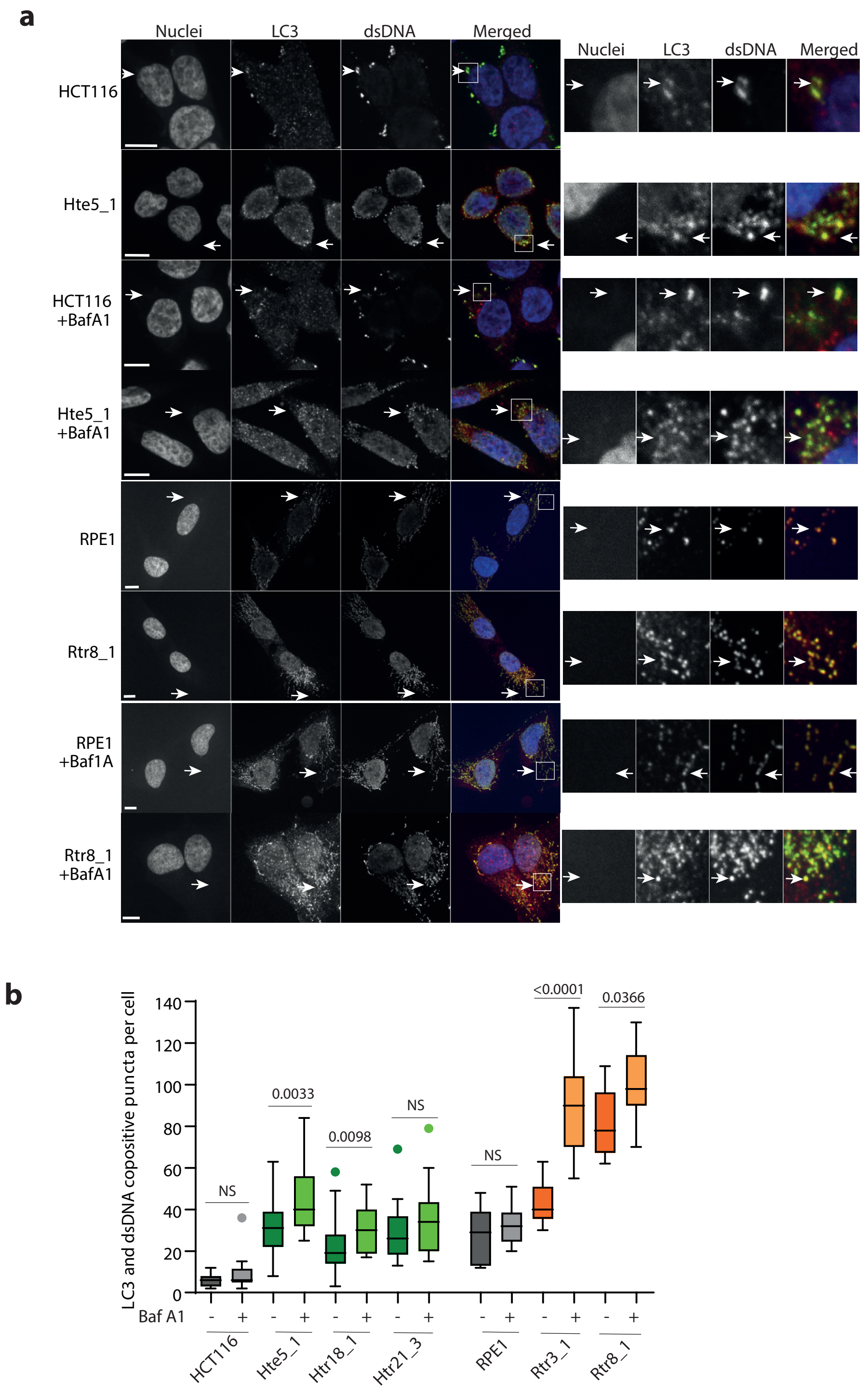

Supplementary Figure 4

dsDNA accumulation in cytoplasm upon autophagy inhibition in trisomic cells. a). Immunofluorescence images of cytoplasmic dsDNA colocolizing with LC3-positive puncta in the presence of Bafilomycin A1 (4 hours). White arrows point on colocalization of dsDNA and LC3. b) Quantification of the numbers of dsDNA with LC3 copositive puncta per cell. At least n=20 cellsfor HCT116 and n=10 cells for RPE1 cell lines were analyzed per each condition. Unpaired t test was applied for statistical analysis. Individual measurements, mean values and standard deviations are shown on the plot. Scale bar in all images 10 μm. Statistical evaluation and source data are summarized in Supplementary table 4 and Supplementary Data 6, respectively.

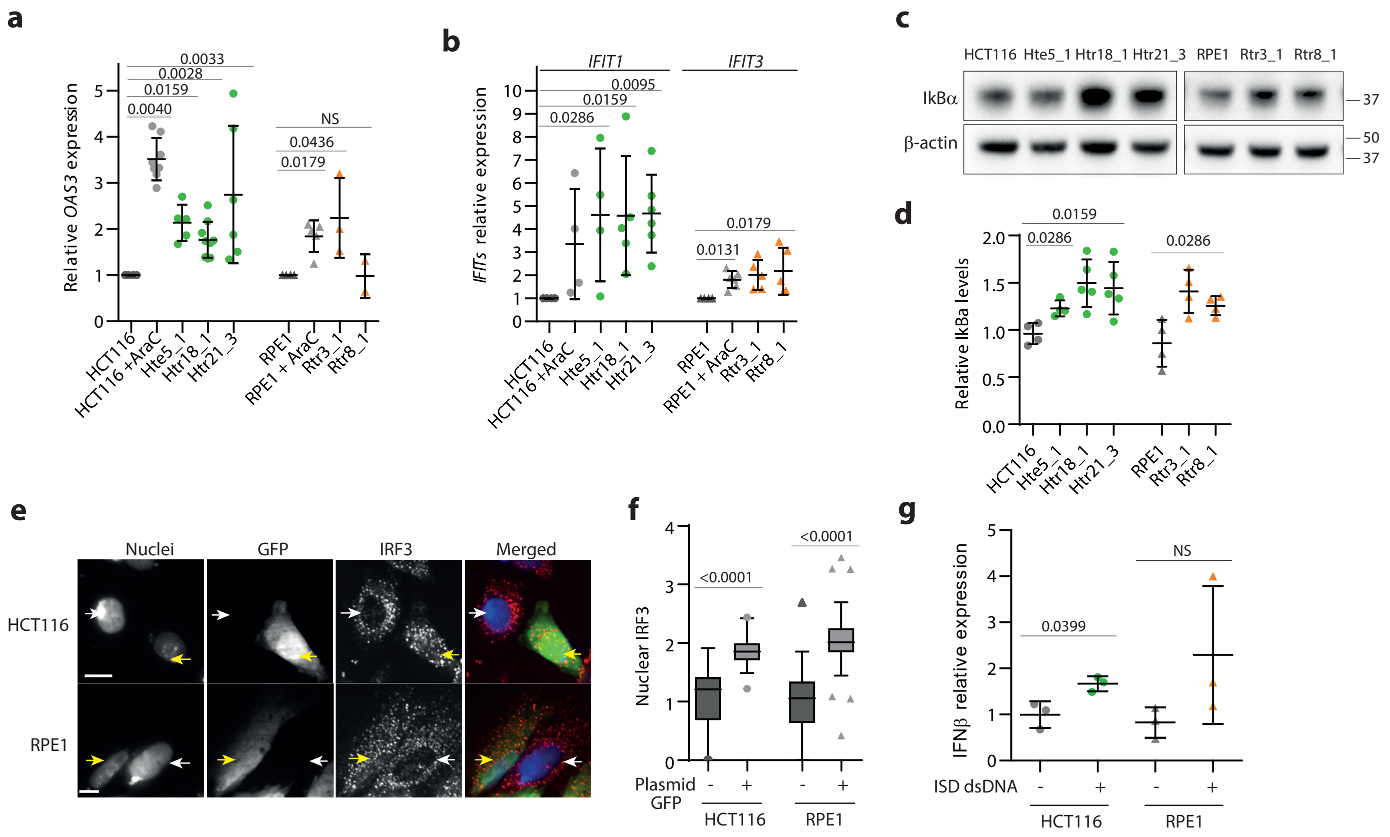

Supplementary Figure 5

Activation of the innate immune pathway in trisomic cells. a), b) Quantification of the relative expression of *OAS3*, *IFIT1* and *IFIT3* evaluated by qRT-PCR. Treatment with AraC was used as a positive control, n=2-9. Immunoblot (c) and relative quantification (d) of IkBα protein, n=4-5. Mann-Whitney test was applied for statistical analysis. Individual measurements, mean values and standard deviations are shown. e) IRF3 localization in cells transfected with a GFP-encoding plasmid. The GFP positive cells show IRF3-positive nuclei (yellow arrows). f) Quantification of nuclear IRF3 intensity in cells from figure (e). At least n=30 cells were analyzed for each condition. Data were normalized to the signal of the control untransfected cells, with IRF3-negative nuclei (white arrows). Unpaired t test was used to analyzed data on the graph f). g) Quantification of *IFNβ* relative expression after ISD DNA oligo transfection, n=3. Two tailed unpaired t test was performed for statsistical analysis. Scale bar in all images 10 μm. Statistical evaluation and source data are summarized in Supplementary table 4 and Supplementary Data 6.

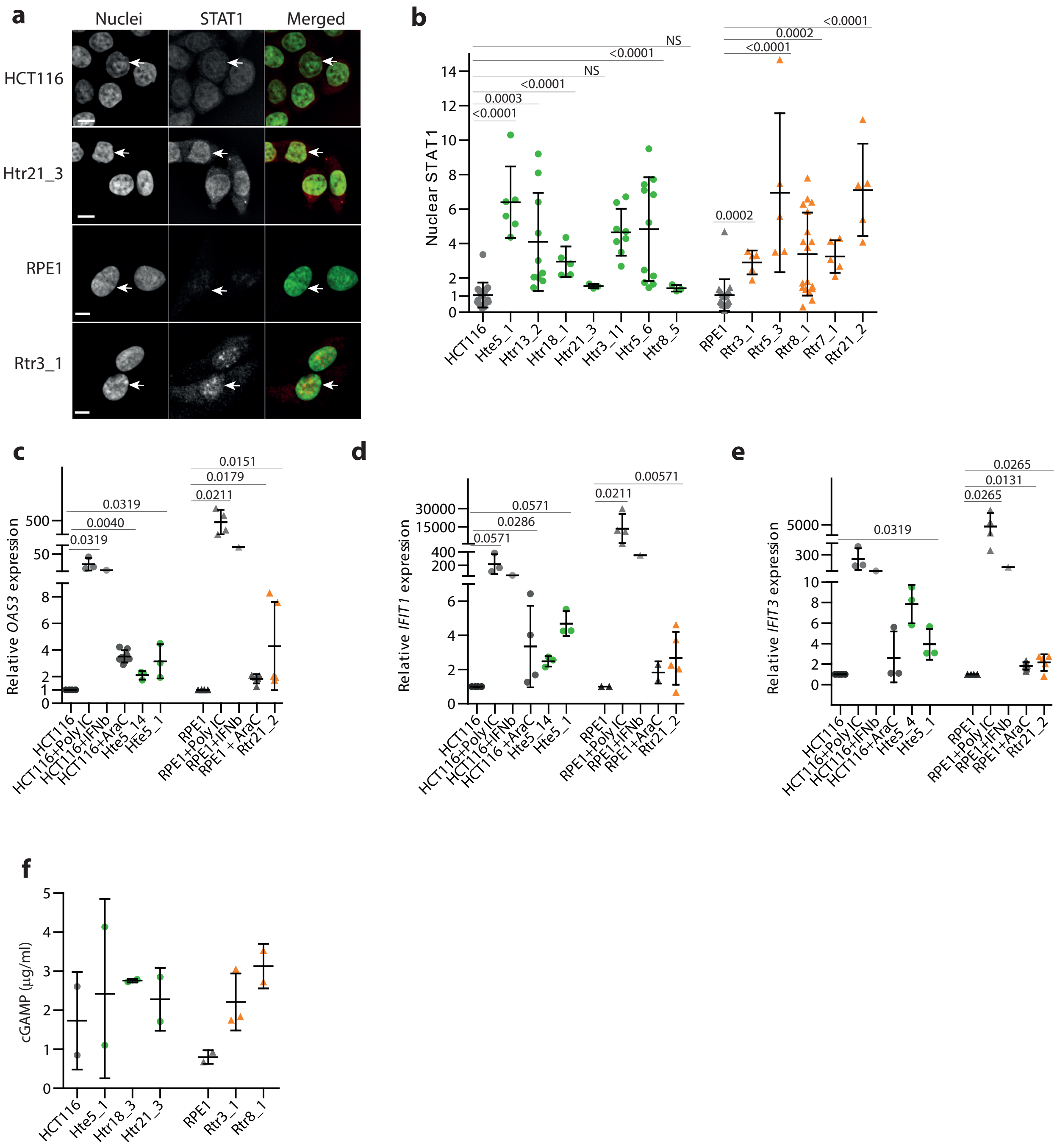

Supplementary Figure 6

Transcriptional activation of the innate immune pathway in trisomic cellsa) Representative images of STAT1 nuclear localization in diploid vs. trisomic cells. b) Quantification of the nuclear STAT1 levels. In average 2000 cells for HCT116 and 1000 cells for RPE1 were analyzed for each cell line. The number of measurements n=3-21 fpr each cell lines. Unpaired t test was used for statistical analysis. c-e) Quantification of the relative expression of *OAS3*, *IFIT1* and *IFIT3* evaluated by qRT-PCR. AraC treatment was used as a positive control. The number of measurements n=3-8 for statistical analysis with Mann-Whitney. h) The levels of cGAMP measured by ELISA. Individual measurements, mean values and standard deviations are shown. Scale bar in all images 10 μm. Statistical evaluation is summarized in Supplementary table 4, source data in Supplementary Data 6.

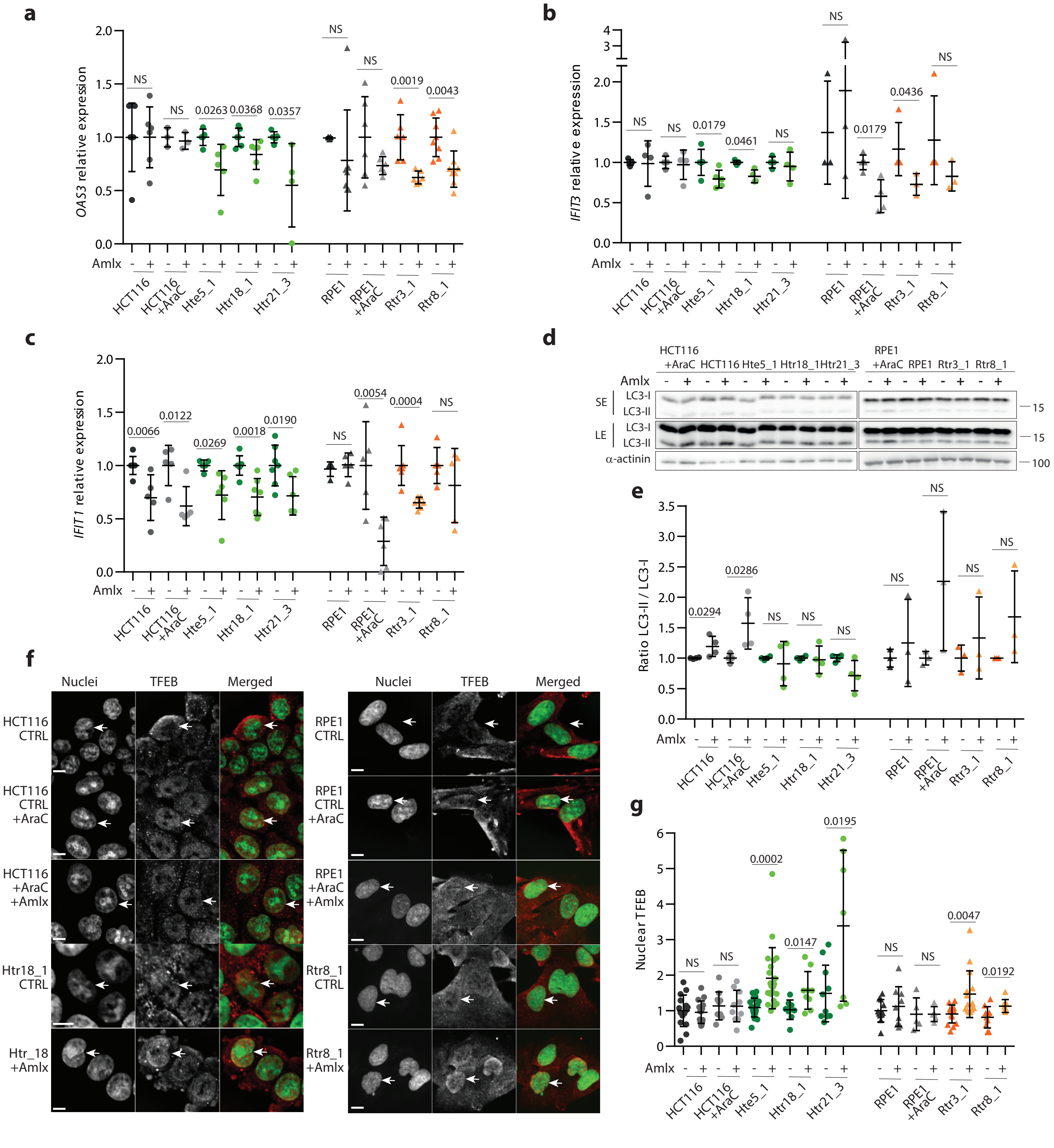

Supplementary Figure 7

Activation of the innate immune response, but not autophagy, depends on TBK1 in constitutive trisomic cells.

a-c) Quantification of relative qRT-PCR of selected IRF3 targets and ISGs after Amlexanox treatment. The data were normalized to the corresponding DMSO controls for each cell line, n=3-7. d) Immunoblotting of LC3-I and LC3-II in diploid and aneuploid cells upon TBK1 inhibition with Amlexanox (Amlx). α-actinin was used as loading control. SE-short exposure, LE - long exposure. e) Quantification of the normalized LC3-II/LC3-I ratio upon TBK1 inhibition with Amlexanox, n=3-4. All samples were normalized to the corresponding DMSO control of every cell line. f) Examples of immunofluorescent images of TFEB localization in cells treated with Amlexanox. White arrows indicate nuclei that are more abundant with TFEB protein with Amlexanox in trisomic cells. g) Quantification of nuclear TFEB signal. In average 1400 cells for HCT116 and 300 cells for RPE1 were analyzed per each cell lines. The number of measurements n=5-9 were performed for statistical analysis with Mann-Whitney test for the graphs b and e. For graphs a, c and g unpaired t test was used to analyze data. Individual measurements, mean and p-values and standard deviations are shown in the plots. Scale bar 10 µm. Statistical evaluation and source data are summarized in Supplementary table 4 and Supplementar Data 6, respectively.

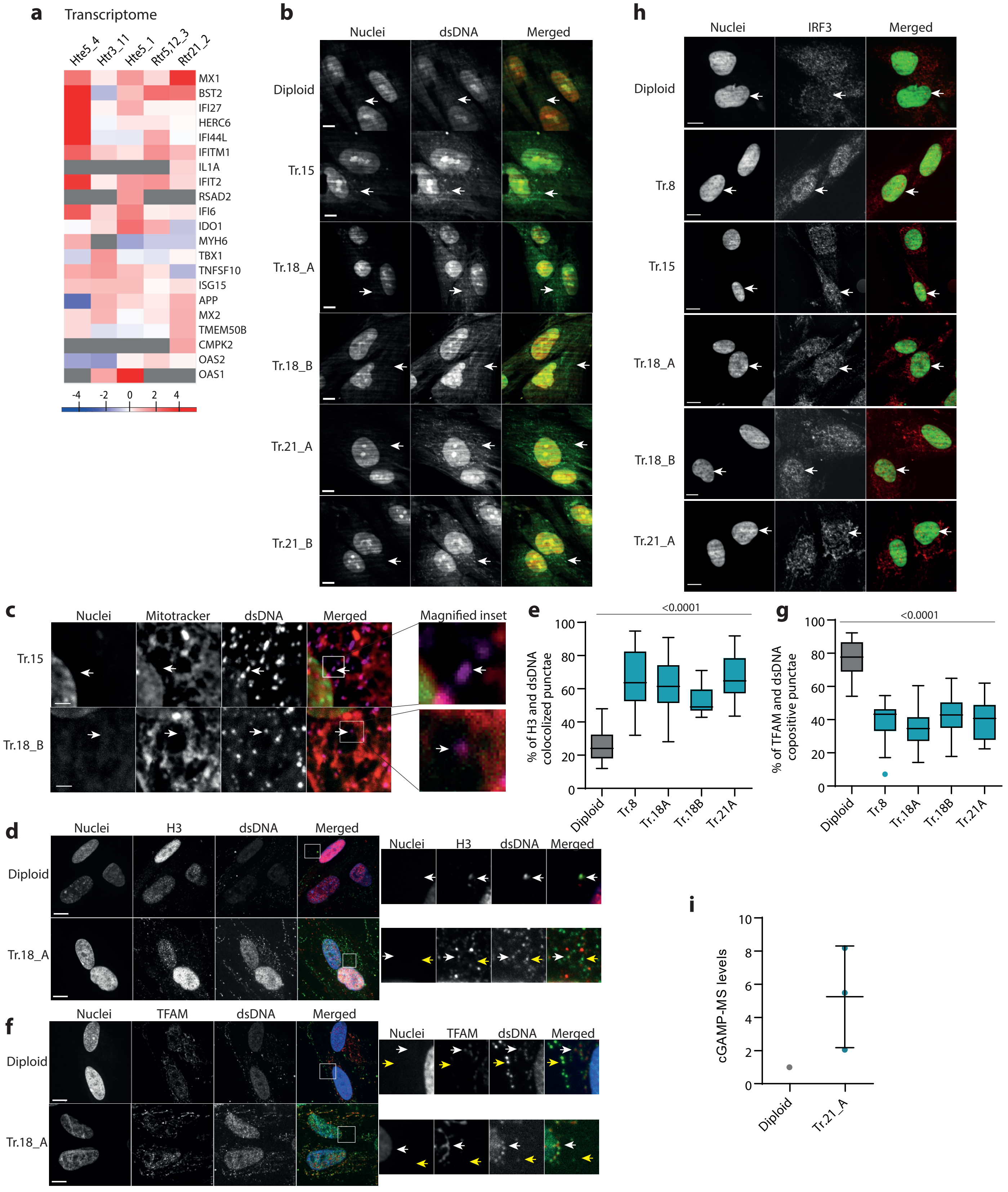

Supplementary Figure 8

Innate immune response in trisomic primary human fibroblasts. a) Heat map based on our transcriptome data shows the expression levels of the inflammatory gene set known to be activated in Down syndrome patients (from Sullivan et al, 2016) in model trisomic cells. b) Representative immunofluorescence images of dsDNA in cytoplasm (white arrows) in primary human fibroblast. c) Representative immunofluorescence images of mitochondria and dsDNA in primary human fibroblasts. White arrows point to colocalization fo dsDNA and mitotracker. Immunofluorescent images of dsDNA and H3 (d) or TFAM (f). White arrows point to dsDNA colocalized with H3 (d) or TFAM (f), yellow arrows indicate absence of colocalization of dsDNA with these markers. Quantification of percentages of dsDNA colocalized with H3 (e) and TFAM (g). In average n=30 cells were analyzed per each cell line. Unpaired t test was applied for data analysis. h) Examples of IF IRF3 images. i) cGAMP levels measured by mass spectrometry. Individual measurements, mean values and standard deviation are shown on the graphs. Scale bar 10 μm. Statistical evaluation and source data are summarized in Supplementary table 4 and Supplementary Data 6.

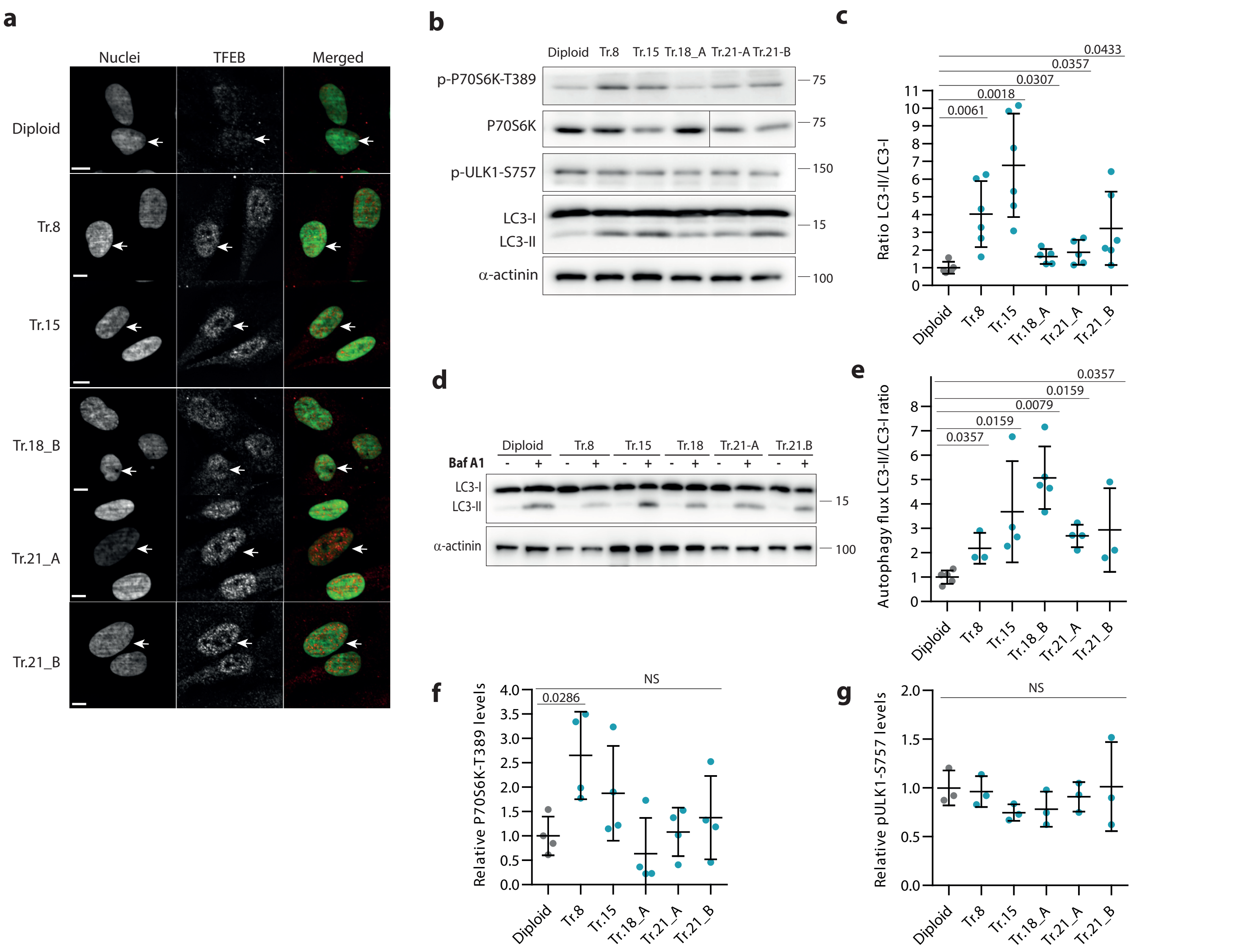

Supplementary Figure 9

Autophagy activity in trisomic primary human fibroblasts a) Localization of TFEB in primary embryonic fibroblasts. White arrows point to the nuclei. Scale bar 10 μm. b) Immunoblotting of mTORC1 downstream phosphorylation targets p-P70S6K-T389, p-ULK1-S757 and LC3 in primary fibroblasts. α-actinin was used as loading control. Quantification of the LC3-II/LC3-I ratio (c, n=5-6) and relative protein levels of p-P70S6K-T389 (f, n=4), p-ULK1-S757 (g, n=3) in primary fibroblast. Data were normalized to diploid control samples. d) Immunoblotting of LC3 upon Bafilomycin 1A treatment (100 nM, 4 hours incubation) in primary fibroblasts. α-actinin was used as loading control. e) Quantification of the LC3II/LC3-I ratio in primary fibroblasts treated with Bafilomycin 1A to estimate the autophagic flux, n=3-5. The LC3-II/LC3-I ratio was calculated similar as in c and normalized to control. Mann-Whitney test was applied for statistical analysis of all graphs, only graph c was analyzed using unpaired t-test. Individual measurements, mean values and standard deviations are shown on the graphs. Statistical evaluation and source data are summarized in Supplementary table 4 and Supplementary Data 6, respectively.
