# Supplementary material for: Genotoxic stress in constitutive trisomies induces autophagy and the innate immune response via the cGAS-STING pathway
Source: Commun Biol. 2021 Jul 2;4:831. doi: 10.1038/s42003-021-02278-9 (PMC8253785; doi:10.1038/s42003-021-02278-9)
Supplement: Supplementary file 2 — Description of Additional Supplementary Files [file 42003_2021_2278_MOESM2_ESM.pdf]

Description of Additional Supplementary Files

File name: Supplementary Data 1

Description: Expression of TFEB-dependent factors in aneuploid cells.

File Name: Supplementary Data 2

Description: Expression of IRF3 and NFkB-dependent factors in aneuploid cells.

File Name: Supplementary Data 3

Description: Expression of NFkB targets evaluated by quantitative rtPCR.

File Name: Supplementary Data 4

Description: Expression of inflammation markers in aneuploid cells.

File Name: Supplementary Data 5

Description: Source data for all main figures.

File Name: Supplementary Data 6

Description: Source data for all supplementary figures.
