# Supplementary material for: Genotoxic stress in constitutive trisomies induces autophagy and the innate immune response via the cGAS-STING pathway
Source: Commun Biol. 2021 Jul 2;4:831. doi: 10.1038/s42003-021-02278-9 (PMC8253785; doi:10.1038/s42003-021-02278-9)
Supplement: Supplementary file 9 — Reporting Summary [file 42003_2021_2278_MOESM9_ESM.pdf]

nature research

Corresponding author(s): Zuzana Storchova

Last updated by author(s): Nov 27, 2020

Reporting Summary

Nature Research wishes to improve the reproducibility of the work that we publish. This form provides structure for consistency and transparency in reporting. For further information on Nature Research policies, see our Editorial Policies and the Editorial Policy Checklist.

Statistics

For all statistical analyses, confirm that the following items are present in the figure legend, table legend, main text, or Methods section.

n/a	Confirmed	
☐	☒	The exact sample size (n) for each experimental group/condition, given as a discrete number and unit of measurement
☐	☒	A statement on whether measurements were taken from distinct samples or whether the same sample was measured repeatedly
☐	☒	The statistical test(s) used AND whether they are one- or two-sided<br>*Only common tests should be described solely by name; describe more complex techniques in the Methods section.*
☒	☐	A description of all covariates tested
☐	☒	A description of any assumptions or corrections, such as tests of normality and adjustment for multiple comparisons
☐	☒	A full description of the statistical parameters including central tendency (e.g. means) or other basic estimates (e.g. regression coefficient) AND variation (e.g. standard deviation) or associated estimates of uncertainty (e.g. confidence intervals)
☒	☐	For null hypothesis testing, the test statistic (e.g. F, t, r) with confidence intervals, effect sizes, degrees of freedom and P value noted<br>*Give P values as exact values whenever suitable.*
☒	☐	For Bayesian analysis, information on the choice of priors and Markov chain Monte Carlo settings
☒	☐	For hierarchical and complex designs, identification of the appropriate level for tests and full reporting of outcomes
☒	☐	Estimates of effect sizes (e.g. Cohen's d, Pearson's r), indicating how they were calculated

Our web collection on statistics for biologists contains articles on many of the points above.

Software and code

Policy information about availability of computer code

Data collection

For immunoblotting, the signal was imaged with the Azure c500 system (Azure Biosystems, Dublin, USA).

Spinning disc confocal laser microscopy was performed using a fully automated Zeiss inverted microscope (AxioObserver Z1) equipped with a MS-2000 stage (Applied Scientific Instrumentation, Eugene, OR), the CSU-X1 spinning disk confocal head (Yokogawa) and LaserStack Launch with selectable laser lines (Intelligent Imaging Innovations, Denver, CO). Image acquisition was performed using a CoolSnap HQ camera (Roper Scientific) and a 20x-air, 40x-air or 63x-oil objective (Plan Neofluar × 40/0.75, Plan Neofluar ×20/0.75) under the control of the SlideBook 6 x64 program (SlideBook Software, Intelligent Imaging Innovations, Denver, CO, USA).

QRT-PCR results were collected with the Bio-Rad CFX.

CellROX data were analysed on a BD FACSCalibur flow cytometer.

For cGAMP mass spec, cell lysates were analyzed by means of LC-MS/MS with a 4000 QTrap (AB Sciex, Darmstadt, 3 Germany) and a Dionex UHPLC UltiMate 3000 (Thermo Fisher) or Prominence HPLC (Shimadzu, 4 Germany).

Data analysis

CellProfiler, a cell image analysis software (https://cellprofiler.org/), was used to quantify microscopy images.

Image J was used to quantify immunoblotting results.

QRT-PCR results were examined with the Bio-Rad CFX Manager.

Bioinformatics analysis of the proteomic and transcriptomic data was performed using Perseus (1.6.2.3) as a part of the MaxQuant Software

Package.

CellROX data were analysed on a BD FACSCalibur flow cytometer.

For manuscripts utilizing custom algorithms or software that are central to the research but not yet described in published literature, software must be made available to editors and reviewers. We strongly encourage code deposition in a community repository (e.g. GitHub). See the Nature Research guidelines for submitting code & software for further information.

Data

Policy information about availability of data

All manuscripts must include a data availability statement. This statement should provide the following information, where applicable:

- Accession codes, unique identifiers, or web links for publicly available datasets
- A list of figures that have associated raw data
- A description of any restrictions on data availability

The transcriptome and proteome data of aneuploid cells that support the findings of this study were uploaded to publicly available databases and are also available in our previously published papers, DOI: 10.1038/msb.2012.40, DOI: 10.1186/1471-2164-15-139.
cGAMP mass spectrometry data are available from the corresponding author upon request.

Field-specific reporting

Please select the one below that is the best fit for your research. If you are not sure, read the appropriate sections before making your selection.

- [x] Life sciences
- [ ] Behavioural & social sciences
- [ ] Ecological, evolutionary & environmental sciences

For a reference copy of the document with all sections, see nature.com/documents/nr-reporting-summary-flat.pdf

Life sciences study design

All studies must disclose on these points even when the disclosure is negative.

Sample size	For immunofluorescent analysis, 100-1000s of cells were analyzed using automatic software. The means of each measurement/experiment were calculated and plotted for presentation in the paper. Samples analyzed manually are labeled in the publication, 20 - 100 cells were analyzed in these experiments. Every point in the plots of data for qRT-PCR, WB or other similar assays analysis represents one individual experiment, total sample size is represented by 3-10 points on the graph.
Data exclusions	Data were not excluded.
Replication	At least 3 replicates were performed; the exact number of replicates is stated in figure legends.
Randomization	Sample and experimental set up was as random as possible.
Blinding	N/A

Reporting for specific materials, systems and methods

We require information from authors about some types of materials, experimental systems and methods used in many studies. Here, indicate whether each material, system or method listed is relevant to your study. If you are not sure if a list item applies to your research, read the appropriate section before selecting a response.

Materials & experimental systems

n/a	Involved in the study	
[ ]	[x]	Antibodies
[ ]	[x]	Eukaryotic cell lines
[x]	[ ]	Palaeontology and archaeology
[x]	[ ]	Animals and other organisms
[x]	[ ]	Human research participants
[x]	[ ]	Clinical data
[x]	[ ]	Dual use research of concern

Methods

n/a	Involved in the study	
[x]	[ ]	ChIP-seq
[x]	[ ]	Flow cytometry
[x]	[ ]	MRI-based neuroimaging

Antibodies

Antibodies used

All the information is provided in Supplementary table 6 of this manuscript. Here we copy it for you.
Antibody Conditions used Reference Company
WB IF

AKT 1:1000 WB 9272 Santa Cruz Biotechnology
p-Akt (Ser473) 1:1000 WB 4060 Cell Signaling Technology
AMPK1 (D5A2) 1:1000 WB5831 Cell Signaling Technology
p-AMPKα-Thr172 1:1000 WB 2531 Cell Signaling Technology
a-actinin (H-2) 1:1000 WB 17829 Santa Cruz Biotechnology
cGAS (D1D3G) 1:1000-WB, 1:100-IF 15102 Cell Signalling Technology
dsDNA 1:50 IF 58749 Santa Cruz Biotechnology
GAPDH 1:1000 WB14C10 Cell Signaling Technology
Histone H3 1:100 IF 9715 Cell Signaling Technology
HRP Mouse 1:5000 WB HAF007 R&D Systems
HRP Rabbit 1:5000 WB HAF008 R&D Systems
H3 1:500 ab12079 IF Abcam
IκBα (L35A5) 1:1000 WB 48144S Cell Signaling Technology
IRF3 (D83B9) 1:50 IF 4302 Cell Signaling Technology
LAMP2 2 μg/mL IF ab13534 Abcam
LC3B (EPR18709) 1:3000 WB 2 μg/ml 192890 Abcam
mTOR (EPR390(N)) 1:500 WB ab134903 Abcam
NRF2 (D1Z9C) XP 1:1000-WB, 1:100-IF 12721 Cell Signaling Technology
P70S6K 1:1000 WB 2708 Cell Signaling Technology
p-P70S6 (Thr389) 1:500 WB 9205 Cell Signaling Technology
STAT1 1:100-IF N9172 Cell Signaling Technology
STING (D2P2F) 1:1000-WB, 1:50-IF 13647S Cell Signaling Technology
p-STING (S366) (D7C3S) 1:1000 WB 19781S Cell Signaling Technology
TBK1 (108A429) 1 μg/mL -WB Ab12116 Abcam
p-TBK1 (Ser172)(D52C2) 1:500 WB 54834 Cell Signaling Technology
TFAM 2 μg/mL IF ab176558 Abcam
TFE3 2 μg/mL-IF HPA023881 Sigma (Atlas antibodies)
TFEB 5 μg/mL-IF MAB9170 R&D systems
p-ULK1 (Ser757) (D7O6U) 1:1000 WB 14202 Cell Signaling
β-Actin-HRP (C4) 1:2500 WB Santa Cruz sc-47778

Validation

For the WB, antibodies were validated based on the appropriate MW of the target protein. Additionally, via immunofluorescence the appropriate intracellular localization and distribution pattern of the protein were noted.

Eukaryotic cell lines

Policy information about cell lines

Cell line source(s)

HCT116 and RPE1 were originally purchased from ATCC. All other cell lines were derived in house and reported in the previous publications from our group.

Authentication

Sequencing, chromosome painting

Mycoplasma contamination

The cell lines were test negative for Mycoplasma contamination.

Commonly misidentified lines
(See ICLAC register)

NA
